# Network‐Informed Gene Ranking Tackles Genetic Heterogeneity in Exome‐Sequencing Studies of Monogenic Disease

**DOI:** 10.1002/humu.22906

**Published:** 2015-10-07

**Authors:** Nick Dand, Reiner Schulz, Michael E. Weale, Laura Southgate, Rebecca J. Oakey, Michael A. Simpson, Thomas Schlitt

**Affiliations:** ^1^ Division of Genetics and Molecular MedicineKing's College LondonLondonUK; ^2^ Barts and The London School of Medicine and DentistryQueen Mary University of LondonLondonUK; ^3^ Institute for Mathematical and Molecular BiomedicineKing's College LondonLondonUK

**Keywords:** whole‐exome sequencing, next generation sequencing, NGS, rare disease, monogenic, Mendelian, genetic heterogeneity, variant prioritization, interaction networks

## Abstract

Genetic heterogeneity presents a significant challenge for the identification of monogenic disease genes. Whole‐exome sequencing generates a large number of candidate disease‐causing variants and typical analyses rely on deleterious variants being observed in the same gene across several unrelated affected individuals. This is less likely to occur for genetically heterogeneous diseases, making more advanced analysis methods necessary. To address this need, we present HetRank, a flexible gene‐ranking method that incorporates interaction network data. We first show that different genes underlying the same monogenic disease are frequently connected in protein interaction networks. This motivates the central premise of HetRank: those genes carrying potentially pathogenic variants and whose network neighbors do so in other affected individuals are strong candidates for follow‐up study. By simulating 1,000 exome sequencing studies (20,000 exomes in total), we model varying degrees of genetic heterogeneity and show that HetRank consistently prioritizes more disease‐causing genes than existing analysis methods. We also demonstrate a proof‐of‐principle application of the method to prioritize genes causing Adams‐Oliver syndrome, a genetically heterogeneous rare disease. An implementation of HetRank in R is available via the Website http://sourceforge.net/p/hetrank/.

## Introduction

It has become standard practice to employ whole‐exome sequencing for the identification of genes causing rare monogenic diseases [Ng et al., 2009; Rabbani et al., 2012]. An efficient and popular strategy is “intersection filtering” [Robinson et al., 2011], in which several unrelated affected individuals are whole‐exome sequenced and their sequence variants filtered and compared. Filtering is necessary to reduce the large number of sequence variants identified for each affected individual [Li et al., 2012], and is achieved by discarding variants which fail to meet specific criteria based on an *a priori* expectation of the genetic architecture of the disease under study. Typically for rare monogenic diseases, discarded variants could include those with minor allele frequencies exceeding a specified threshold, with a predicted mild functional impact, or which have been observed in an unaffected control dataset [Bamshad et al., 2011; Robinson et al., 2011; Li et al., 2012]. Subsequently, genes in which a large proportion of the affected individuals carry a post‐filtering variant are strong candidates for disease causality and can be validated (or rejected) by other methods [Hood et al., 2012; Jones et al., 2012; Lines et al., 2012; Nakazawa et al., 2012; Polvi et al., 2012; Simpson et al., 2012].

This approach can be applied to both rare inherited and sporadic disorders, and does not require prior knowledge of the disease process or a set of candidate genes. However, while the search is genome wide, intersection filtering is attractive because the number of genes in which several unrelated individuals carry post‐filtering variants will generally be small. Conversely, the effectiveness of the approach can be limited by missing data, non‐exonic causal variants, or genetic (locus) heterogeneity [Robinson et al., 2011; Boycott et al., 2013]. In this work, we focus in particular on genetic heterogeneity, that is, mutations occurring in different genes causing the same phenotypic outcome in different patients—thus, intersection filtering would likely fail to reveal those genes [Oti and Brunner, 2007; McClellan and King, 2010].

We present here a flexible analysis approach (“HetRank”) that addresses the problem of genetic heterogeneity in exome sequencing studies by incorporating information from biological networks (such as protein interaction networks). Networks are ideally suited to this purpose because biological function is hypothesized to arise from systems of molecular interactions [Emmert‐Streib and Glazko, 2011]. Networks that systematically describe these interactions can therefore group together functionally related genes beyond existing curated biological pathways [Lehne and Schlitt, 2012]. Finally, the protein products of genes causing phenotypically similar diseases are more likely to physically interact than those from non‐disease genes [Goh et al., 2007; Feldman et al., 2008]. The use of interaction data is also intuitively reasonable at a molecular level: each partner in a molecular interaction is vulnerable to mutation and a disease phenotype may result from a mutation disrupting the interaction and thus, the cooperatively achieved function [Barabasi et al., 2011; Vidal et al., 2011].

We previously presented BioGranat‐IG [Dand et al., 2013], a software tool that searches biological networks for candidate disease‐causing pathways: small sets of genes whose connectedness in the network implies a functional relationship, and which when taken together carry a post‐filtering sequence variant for all (or most) of the patients in an exome‐sequencing study. HetRank has several advantages over BioGranat‐IG. By incorporating network information into a gene‐ranking framework, HetRank retains the ability to prioritize genes that are not included in the chosen input network. It also deals explicitly with the problem caused by highly connected (hub) genes in the network, which in practice can occur frequently in BioGranat‐IG results because of their connectivity rather than true disease involvement. The flexible ranking framework allows incorporation of diverse sources of information for variant prioritization, and limits the risk of excluding true disease‐causing variants due to hard filtering thresholds. Finally, HetRank can incorporate healthy control exomes to address the overrepresentation of long and variant‐tolerating genes among prioritized variants, a common problem in exome sequencing studies [Fuentes Fajardo et al., 2012; Petrovski et al., 2013].

There exist several variant prioritization tools appropriate for the study of rare monogenic diseases that integrate various sources of evidence for causality [Li et al., 2012; Sifrim et al., 2012; Carter et al., 2013; Frousios et al., 2013; Sifrim et al., 2013; Robinson et al., 2014]. However, where interaction data are used, it is generally either to prioritize genes based on their proximity to known disease genes in the interaction network, or to allow the user to explore genes which interact with those prioritized. To our knowledge, HetRank is the first approach to incorporate interaction network information directly into the gene‐ranking procedure in a hypothesis‐free manner as a means of addressing genetic heterogeneity.

We test our new approach using a set of test data comprising 20,000 simulated exomes derived from real exomes and show here that network information helps to rank disease‐causing genes highly even under conditions of high genetic heterogeneity. Further, we have applied HetRank to the prioritization of known disease‐causing genes in Adams‐Oliver syndrome (AOS; MIM# 100300), a rare developmental disorder that can be caused by variants in several different genes [Stittrich et al., 2014].

## Materials and Methods

All data analysis is performed in the R programming language [R Development Core Team, 2013], using the igraph [Csardi and Nepusz, 2006] library for network analysis. We make available online an implementation of HetRank in R (http://sourceforge.net/p/hetrank).

### Protein Interaction Networks and Disease Subnetworks

To justify the use of interaction networks to address genetic heterogeneity, we investigated the extent to which genes causing the same disease interact in a protein interaction network. Protein–protein interaction data were obtained from the Protein Interaction Network Analysis platform, a meta‐database derived from six manually curated interaction databases [Cowley et al., 2012] (downloaded 20th December 2012) and used to construct undirected and unweighted networks of binary interactions. *PINA* is the network containing all interactions (14,434 genes, 105,801 interactions) and *PINAmin2* is a high‐confidence subnetwork consisting of only those interactions that have two or more different publication identifiers in the database (7,417 genes, 18,092 interactions).

Disease‐gene mappings were obtained from OMIM's Morbid Map (downloaded 20th March 2013). Unconfirmed mappings and mappings that either involve non‐disease phenotypes or where the gene is not directly causal (such as genes which contribute to susceptibility to a multifactorial disorder or infection) were excluded, leaving 4,956 mappings. OMIM disease terms were replaced with generalized disease terms by removing disease “type” or “group” names, and any words consisting entirely of numbers. This resulted in a set of 3,193 generalized disease terms, 541 of which displayed genetic heterogeneity by mapping to more than one causal gene. In each protein interaction network, disease subnetworks were found by considering one generalized disease term at a time and identifying direct interactions between causal genes.

To estimate the likelihood that disease subnetworks could arise by chance due to the number of disease genes mapping into each network, the same procedure was also performed for 10,000 randomly permuted networks. In order to counter the potential bias in curated interaction networks toward well‐studied disease‐causing genes, we performed degree‐constrained permutation tests for each network to test the null hypothesis (*H*_0_) that the number of observed disease subnetworks is a consequence of the number and degree distribution of disease‐causing genes in the network, against the null hypothesis (*H*_1_) that we observe more disease subnetworks than could result from number and degree distribution alone (Supp. Methods).

### HetRank Gene Prioritization Approach

**Figure 1:**
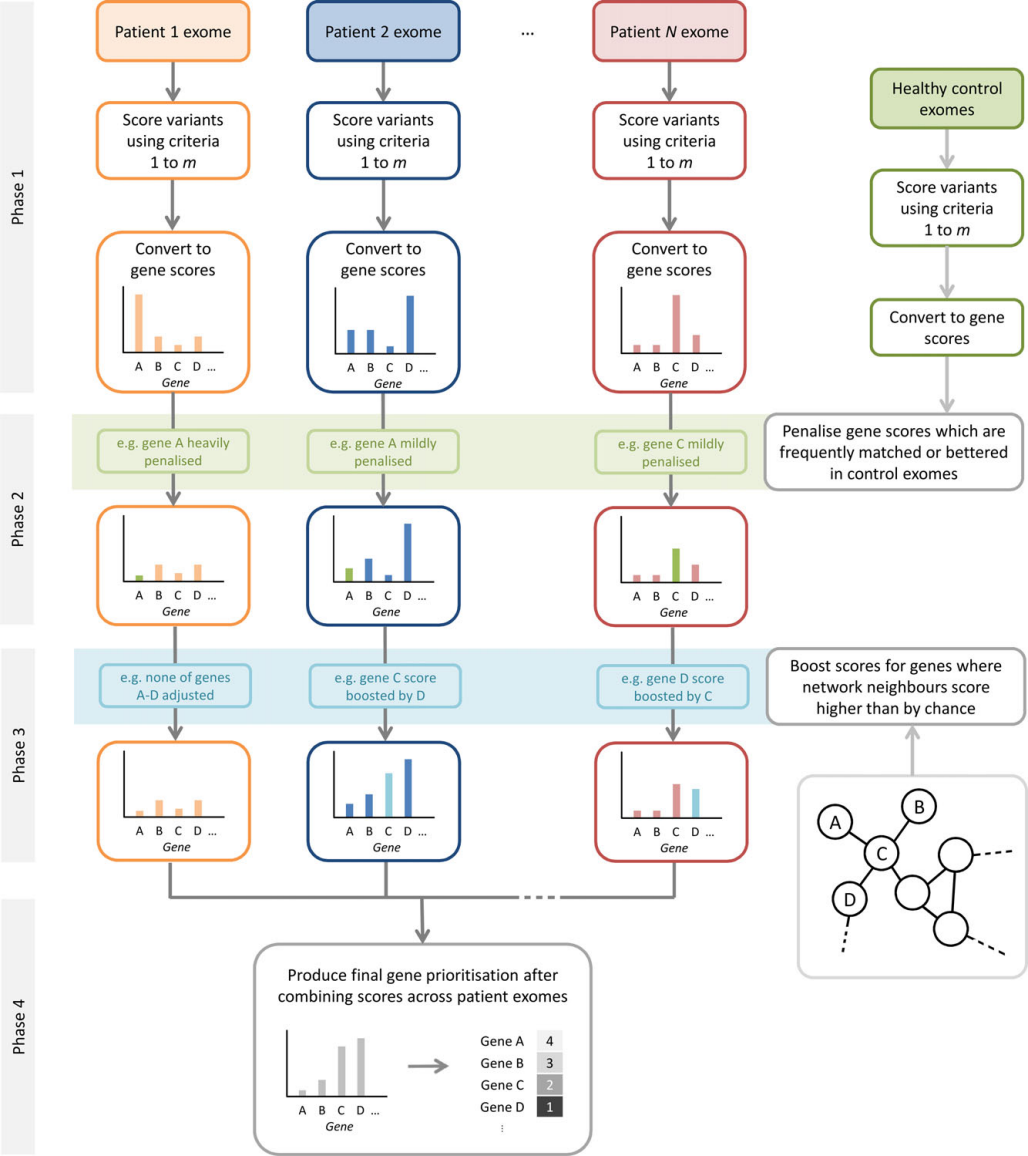
The HetRank analysis framework. Phase 1: Variants are ranked in each affected individual's exome sequence according to a set of user‐specified criteria and converted into gene scores. Phase 2: Gene scores are adjusted with respect to the scores achieved in a set of healthy control exomes, allowing more accurate prioritization of long and variant‐tolerating genes. Phase 3: An interaction network is used to share score information between neighboring genes. Neighboring genes that score highly in different affected individuals improve the evidence for one another's involvement in the disease process. Phase 4: Scores are combined across all exomes in the study to give a final prioritized list of genes.

We subsequently developed a method to prioritize genes for follow‐up analysis in exome sequencing studies of monogenic disease, using an interaction network to overcome genetic heterogeneity (Fig. 1). Briefly, sequence variants identified in the exomes of a number of unrelated affected individuals are independently scored (according to evidence supporting each variant's disease involvement), and a final prioritization of genes is produced by combining scores across all exomes in the study. The final gene ranking takes into account variants found in neighboring genes in the interaction network, allowing genes which do not initially score highly in a given individual to have their score improved based on evidence for disease involvement from a neighboring gene; this preferentially improves the final ranking of disease genes because the sharing of evidence between any pair of interaction partners is more likely to occur consistently across unrelated individuals as a result of true genetic heterogeneity than due to chance. The four key phases are as follows.

Phase 1: for each of *N* affected individuals in the study, generate gene scores according to evidence for disease involvement. Scores are derived from variant annotations thought to be informative regarding the likelihood of a variant causing the disease being studied. Intersection filtering would remove variants from further consideration by applying hard thresholds to these annotations; here they are used as ranking criteria. Such criteria typically include the alternative allele frequency (effectively set to zero for novel sequence variants) and variant effect (e.g., synonymous, missense, nonsense). As ranking criteria are specified by the user, functional prediction scores or quantification of disease‐specific knowledge may also be included. With respect to each annotation in turn, all variants in an exome are ranked, with average rank being used to resolve ties. User‐supplied reference tables allow ranking of ordered categorical criteria. This rank is transformed into a score by taking its reciprocal and scaling: the reciprocal rank is a decaying function which prevents overweighting of variants with little evidence for disease involvement; the scaling ensures that when there are multiple joint top‐ranked variants, all are assigned the maximum score of 1. To incorporate user expectations of plausible disease‐causing variants, a “filter‐equivalent” value for the annotation can be supplied (the same value that would be used as a threshold for intersection filtering), and the scores are scaled so that variants ranking better than this value score ≥0.5 (Supp. Fig. S1). A variant's final score is calculated by summing the scores it achieves across all *m* ranking criteria. Finally, gene scores are calculated based on the mode of inheritance assumed. In autosomal dominant (AD) mode HetRank assumes one heterozygous variant is sufficient to cause the disease, and gene scores equate to the maximum score of any heterozygous variant they contain. In autosomal recessive (AR) mode, gene scores are either the highest score of a homozygous variant or half the sum of the top two heterozygous variants, whichever is greater (this accounts for the possibility of compound heterozygous disease‐causing mutations). In neutral mode, gene scores are set to the maximum score of any variant they contain, regardless of zygosity. At the end of phase 1, each gene has received a score for each of the *N* exomes, the highest score achievable being *m*, and the lowest being 0 (where no variant is found, corresponding to no evidence for disease involvement).

Phase 2: use a set of unaffected control exomes to penalize long and variant‐tolerating genes that might otherwise receive an artificially high ranking. To facilitate this, the gene‐scoring procedure described in phase 1 is repeated for each control exome. For affected individual *i*, suppose that gene *g* has a score of $S_{g}$ after phase 1. Now this score is adjusted to $S_{g}^{{}^{\prime }}=S_{g}/\left(1+K\right)$, where *K* is the number of control exomes in which gene *g* has a score $\geq S_{g}$. Thus, the adjustment reflects how frequently variants observed in gene *g* in unaffected controls show evidence for disease causality that is at least as great as that observed in the affected individual (based on the ranking criteria used). This construction means that gene *g*’s score for exome *i* remains unchanged if it is not matched or surpassed in any control exome, and that higher numbers of control exomes should allow greater potential to eliminate frequently mutated genes.

Phase 3: further adjust gene scores with respect to network interactions for each of the *N* individuals. The key assumption of our approach is that in the context of genetic heterogeneity, evidence for the pathogenicity of a gene as part of a functional pathway encoded by network interactions can arise from a plausible sequence variant in the gene itself, or in a gene with which it interacts. This motivates the network adjustment, in which a gene's score can be superseded by the higher score of a neighboring gene, *provided the better score is unlikely to have been observed by chance* (e.g., because the gene has many neighbors in the network). For a gene *g*, the network *d‐*neighborhood, $N_{d}\left(g\right)$, is the set of genes which can be reached from *g* via *d* interactions or fewer (that is, whose distance from *g* in the network is ≤*d*; this always includes *g* itself). Suppose that gene *h* has the highest score ($S_{h}^{{}^{\prime }}$) of all genes in $N_{d}\left(g\right)$ after phase 2. By considering the distribution of scores among all genes in the network, we can establish the probability *p* that a set of $|N_{d}\left(g\right)|$ randomly chosen genes in the network includes a gene with score $\geq S_{h}^{{}^{\prime }}$. If *p* < 0.1, we consider that gene *g* is unlikely to have a neighbor score as highly as gene *h* by chance, and propose the adjusted score $S_{g}^{\left(d\right)}=S_{h}^{{}^{\prime }}/\left(1+d\right)$. Note that the score is penalized via the denominator to reflect the indirect nature of the evidence provided by *h* to support *g* being disease causing. Conversely if *p* ≥ 0.1, we propose no adjustment to the score, so that $S_{g}^{\left(d\right)}=\;0$. As disease subnetworks are generally expected to be small and for reasons of computational feasibility we consider *d*‐neighborhoods up to *d* = 2 and set the network‐adjusted score for gene *g* to be $S_{g}^{{}^{\prime \prime }}=\max\left(S_{g}^{{}^{\prime }},S_{g}^{\left(1\right)},S_{g}^{\left(2\right)}\right)$. For each affected individual, gene scores still range between 0 and *m* at the end of phase 3.

Phase 4: sum network‐adjusted gene scores across the *N* individuals in the study. Each gene therefore has a final score between 0 and *m* × *N*. Genes can be prioritized for follow‐up study by ranking according to these final scores.

### Simulation of Exome Sequencing Studies

To measure the performance of our approach, we simulated 1,000 exome sequencing studies for rare monogenic disease (Supp. Fig. S2). Each study was “spiked” with disease‐causing variants chosen to model genetic heterogeneity, and we tested HetRank's ability to recover the spiked genes.

Chromosome‐wise random selection (without replacement) of sequence data from 388 exomes obtained through the rare disease programme at King's College London (KCL) was used to generate 200 test exomes. These were partitioned into 10 sets of 20 exomes so that when each set was used to simulate an exome sequencing study the other 180 exomes were available to represent healthy controls. This process was repeated 100 times to generate 1,000 simulated exome sequencing studies (20,000 random exomes in total). The KCL exomes were ascertained from unrelated individuals and include some small groups of patients sharing a rare disease phenotype, although the randomization used should be sufficient to overcome any disease‐specific bias in the test data set. Exome variants were annotated using ANNOVAR [Wang et al., 2010] to give the following variant‐ranking criteria: variant effect (e.g., “nonsynonymous SNV”, “stopgain SNV”), Exome Variant Server (EVS) alternative allele frequency, 1000 Genomes Project alternative allele frequency (Supp. Fig. S3).

To simulate exome sequencing studies, a disease‐causing mutation was added to each of the 20 exomes in a set. These were randomly selected from 13,413 pathogenic variants in dbSNP [Sherry et al., 2001] (build 138, downloaded 19th July 2013) that corresponded to monogenic diseases in OMIM's Morbid Map [Amberger et al., 2009] and were annotated using the same pipeline as the KCL exome data. As dbSNP variants did not include zygosity this was assigned according to the mode of inheritance being simulated. Thus, when testing an AR disease model, with probability 0.9 case exomes were equally likely to be spiked with one homozygous variant or two heterozygous variants (corresponding to a compound heterozygous disease‐causing mutation); with probability 0.1 they would be assigned one heterozygous variant. Under an AD disease model the probabilities are switched so that a single heterozygous variant is added with probability 0.9. The small “error” probability of 0.1 is a modest allowance accounting for real‐world uncertainties such as zygosity‐calling errors during sequencing or incorrectly inferred modes of inheritance in sequenced affected individuals.

Gene names for the disease‐causing variants were replaced in order to model genetic heterogeneity. For this purpose, OMIM disease subnetworks were randomly selected from those identified in PINA and PINAmin2 (Supp. Table S1). PINA contains 305 unique disease subnetworks of two genes (including gene pairs drawn from larger disease subnetworks), 248 of three genes and 280 of four genes; PINAmin2 contains 150 of two genes, 108 of three genes and 111 of four genes. The spiked disease‐causing variants were assigned gene names from the disease subnetwork with probability 1‐*u* (captured heterogeneity) or a gene name selected uniformly at random from the whole exome with probability *u* (uncaptured heterogeneity). Uncaptured heterogeneity was modeled to account for any proportion of disease cases explained by reasons other than a mutation in the disease subnetwork. To simulate balanced captured heterogeneity gene names from the disease subnetwork were randomly selected with equal probability. To simulate unbalanced captured heterogeneity gene names from the disease subnetwork were used with probabilities *p*_1_ = 3(1 − *u*)/4, *p*_2_ = (1 − *u*)/4 for two‐gene disease subnetworks; *p*_1_ = (1 − *u*)/2, *p*_2_ = *p*_3_ = (1 − *u*)/4 for three‐gene disease subnetworks, and *p*_1_ = (1 − *u*)/2, *p*_2_ = *p*_3_ = *p*_4_ = (1 − *u*)/6 for four‐gene disease subnetworks.

### HetRank Parameters for Testing

All testing of the simulated exome sequencing studies was performed using PINAmin2 as the input network for HetRank. The three ranking criteria used were EVS and 1000 Genomes alternative allele frequencies, and variant effect (truncating or splicing variants > protein‐altering variants > synonymous variants). The filter‐equivalent threshold values used were 0.1% for the allele frequencies and protein‐altering for variant effect.

### Ranking Based on Intersection Filtering

HetRank results were compared against those achieved by intersection filtering, obtained as follows. For each simulated exome sequencing study, gene lists for intersection filtering were generated for each individual by excluding synonymous variants and variants with EVS or 1000 Genomes alternative allele frequency >0.1%. Analogous to HetRank's AD, AR and neutral modes, gene lists could be further filtered by zygosity (by excluding homozygous variants in AD mode; by requiring genes to contain one homozygous or two heterozygous variants after filtering in AR mode). An additional gene‐wise filtering step was performed by excluding genes which contain post‐filtering sequence variants for five or more of 180 healthy control exomes. These filtering steps were determined to give the best performance for the intersection filtering method through testing on simulated data (Supp. Methods and Supp. Table S2).

To obtain a ranking for potential disease involvement based on intersection filtering, genes were ranked according to the number of filtered gene lists in which they appear, with average rank being used to resolve ties. Genes that are excluded or carry no post‐filtering variants in a study were assigned a default rank of 10,000.

### BioGranat‐IG Comparison

To test whether our new approach improves upon BioGranat‐IG [Dand et al., 2013], BioGranat‐IG was run using the same gene lists as derived for intersection filtering, described above. The best‐performing BioGranat‐IG settings were determined through testing on simulated data (Supp. Methods and Supp. Table S3). Thus, BioGranat‐IG triplet search was run using default settings (results flexibility parameters set to zero) and with a hub‐free version of PINAmin2 as the input network (obtained by excluding genes with 50 or more interaction partners).

### Application to AOS Exomes

To test the ability of HetRank to prioritize the AOS‐causing genes *NOTCH1* and *DLL4*, whole‐exome sequencing data was used from 13 probands affected with an AD form of AOS. Sequencing was performed at KCL and has been described previously [Meester et al., 2015; Southgate et al., 2015]. Within this cohort, two cases harbor truncating *NOTCH1* mutations (1‐II‐1 and 2‐II‐1) and one has a missense *DLL4* variant (case 6‐II‐1), respectively. For unaffected control exomes, we used 346 of the 388 KCL exomes, which do not have an AOS phenotype. HetRank was run in AD mode with parameters as previously described.

## Results

### Interacting Genes Cause the Same Monogenic Diseases

We found 172 connected subnetworks in the PINA network, and 84 in the PINAmin2 network, each of which is causal for a single disease (Table 1; Supp. Table S1). Using a degree‐constrained method of permutation to test the null (*H*_0_) versus alternative hypothesis (*H*_1_) of whether the number and degree distribution of disease‐causing genes account for the number of disease subnetworks, in each case the observed number of disease subnetworks is highly significant (*P* < 10^−4^; Supp. Fig. S4), leading us to reject the null (*H*_0_) and supporting previous assertions that interacting genes are more likely to have similar phenotypic consequences [Goh et al., 2007; Feldman et al., 2008]. This makes a compelling case for the use of interaction networks as a means to identify new sources of genetic heterogeneity, particularly given that high‐throughput methods are continually improving network coverage [Yu et al., 2011].

**Table 1:** OMIM Disease Subnetworks

	Number of disease subnetworks			
	PINA network		PINAmin2 network	
Size of disease		Permutation		Permutation
subnetwork	Observed	average	Observed	average
2	124	12.54 ± 3.35	61	3.46 ± 1.93
3	27	1.65 ± 1.25	11	0.44 ± 0.73
4	11	0.40 ± 0.60	7	0.06 ± 0.25
5+	10	0.28 ± 0.49	5	0.07 ± 0.26
Total	172	14.87 ± 3.46	84	4.03 ± 2.13

Observed = number of disease subnetworks of given size induced by a single disease term in the original network; Permutation average = average number of disease subnetworks of given size induced by a single disease term across 10,000 randomly permuted networks (mean ± standard deviation).

### Network Information Can Improve Ranking of Disease Genes

For each mode of operation (AD, AR, or neutral), HetRank was tested using 1,000 simulated exome sequencing studies. To model genetic heterogeneity, each study was “spiked” with a disease‐causing variant in a gene from an OMIM disease‐specific subnetwork of fixed size (selected at random from those identified in Table 1). For a disease subnetwork of three genes, *g*_1_, *g*_2_, and *g*_3_, each exome in the study would be assigned gene *g_i_* with probability *p_i_*, or a uniformly selected gene from outside the disease subnetwork with probability *u* that represents uncaptured heterogeneity (such that *p*_1_ + *p*_2_ + *p*_3_ + *u* = 1). For each study of 20 exomes there are 180 exomes available to act as healthy controls, with which no sequence data is shared.

Tables 2a and 2b show the results of testing our approach using disease subnetworks of three genes with *u* = 0.5 and balanced captured heterogeneity (*p*_1_ = *p*_2_ = *p*_3_). HetRank's AD and neutral modes were tested with data that simulated an AD mode of inheritance, whereas AR mode was tested with data simulating an AR mode of inheritance. All testing was performed using the high‐confidence PINAmin2 interaction network to inform gene rankings. We measure the performance of our approach by its ability to assign high ranks to the three disease subnetwork genes. This is compared against the performance achieved using a simple intersection filtering approach, using appropriate variant‐ and gene‐filtering criteria and in each study ranking genes according to the number of individuals in which they carry a post‐filtering variant.

**Table 2:** Ability to Recover Disease Subnetworks Comprising Three Genes

	Intersection filtering			HetRank		
	Gene 1	Gene 2	Gene 3	Gene 1	Gene 2	Gene 3
(a)	**Number of 1000 simulations achieving given ranking using high‐coverage disease subnetworks**					
Autosomal dominant mode						
Ranked #1	293			402		
Ranked #1–2	|‐ ‐ ‐ ‐ ‐ ‐ 42 ‐ ‐ ‐ ‐ ‐ ‐|			|‐ ‐ ‐ ‐ ‐ ‐ 201 ‐ ‐ ‐ ‐ ‐ ‐|		
Ranked #1–3	|‐ ‐ ‐ ‐ ‐ ‐ ‐ ‐ ‐ ‐ ‐ ‐ ‐ 3 ‐ ‐ ‐ ‐ ‐ ‐ ‐ ‐ ‐ ‐ ‐ ‐ ‐|			|‐ ‐ ‐ ‐ ‐ ‐ ‐ ‐ ‐ ‐ ‐ ‐ 79 ‐ ‐ ‐ ‐ ‐ ‐ ‐ ‐ ‐ ‐ ‐ ‐|		
Ranked ≤10	590	156	16	649	447	214
Ranked ≤100	787	345	58	838	711	506
Median rank	3	189	10,000	3	16	95.5
Autosomal recessive mode						
Ranked #1	500			901		
Ranked #1–2	|‐ ‐ ‐ ‐ ‐ ‐ 124 ‐ ‐ ‐ ‐ ‐ ‐|			|‐ ‐ ‐ ‐ ‐ ‐ 769 ‐ ‐ ‐ ‐ ‐ ‐|		
Ranked #1–3	|‐ ‐ ‐ ‐ ‐ ‐ ‐ ‐ ‐ ‐ ‐ ‐ ‐ 10 ‐ ‐ ‐ ‐ ‐ ‐ ‐ ‐ ‐ ‐ ‐ ‐ ‐|			|‐ ‐ ‐ ‐ ‐ ‐ ‐ ‐ ‐ ‐ ‐ ‐ ‐ 398 ‐ ‐ ‐ ‐ ‐ ‐ ‐ ‐ ‐ ‐ ‐ ‐ ‐|		
Ranked ≤10	905	504	82	964	911	733
Ranked ≤100	985	830	317	993	976	925
Median rank	1.25	8	243	1	2	4
Neutral mode						
Ranked #1	342			425		
Ranked #1–2	|‐ ‐ ‐ ‐ ‐ ‐ 54 ‐ ‐ ‐ ‐ ‐ ‐|			|‐ ‐ ‐ ‐ ‐ ‐ 229 ‐ ‐ ‐ ‐ ‐ ‐|		
Ranked #1–3	|‐ ‐ ‐ ‐ ‐ ‐ ‐ ‐ ‐ ‐ ‐ ‐ ‐ 5 ‐ ‐ ‐ ‐ ‐ ‐ ‐ ‐ ‐ ‐ ‐ ‐ ‐|			|‐ ‐ ‐ ‐ ‐ ‐ ‐ ‐ ‐ ‐ ‐ ‐ ‐ 83 ‐ ‐ ‐ ‐ ‐ ‐ ‐ ‐ ‐ ‐ ‐ ‐ ‐|		
Ranked ≤10	618	168	20	664	468	236
Ranked ≤100	804	361	73	845	725	523
Median rank	2.5	188.5	10,000	2	13	88
(b)	**Number of 1000 simulations achieving given ranking using low‐coverage disease subnetworks**					
Autosomal dominant mode						
Ranked #1	299			368		
Ranked #1–2	|‐ ‐ ‐ ‐ ‐ ‐ 31 ‐ ‐ ‐ ‐ ‐ ‐|			|‐ ‐ ‐ ‐ ‐ ‐ 124 ‐ ‐ ‐ ‐ ‐ ‐|		
Ranked #1–3	|‐ ‐ ‐ ‐ ‐ ‐ ‐ ‐ ‐ ‐ ‐ ‐ ‐ 1 ‐ ‐ ‐ ‐ ‐ ‐ ‐ ‐ ‐ ‐ ‐ ‐ ‐|			|‐ ‐ ‐ ‐ ‐ ‐ ‐ ‐ ‐ ‐ ‐ ‐ ‐ 36 ‐ ‐ ‐ ‐ ‐ ‐ ‐ ‐ ‐ ‐ ‐ ‐ ‐|		
Ranked ≤10	601	150	5	595	312	115
Ranked ≤100	772	348	52	803	573	293
Median rank	3.5	195	10,000	4	56	456
Autosomal recessive mode						
Ranked #1	496			852		
Ranked #1–2	|‐ ‐ ‐ ‐ ‐ ‐ 138 ‐ ‐ ‐ ‐ ‐ ‐|			|‐ ‐ ‐ ‐ ‐ ‐ 567 ‐ ‐ ‐ ‐ ‐ ‐|		
Ranked #1–3	|‐ ‐ ‐ ‐ ‐ ‐ ‐ ‐ ‐ ‐ ‐ ‐ ‐ 11 ‐ ‐ ‐ ‐ ‐ ‐ ‐ ‐ ‐ ‐ ‐ ‐ ‐|			|‐ ‐ ‐ ‐ ‐ ‐ ‐ ‐ ‐ ‐ ‐ ‐ ‐ 209 ‐ ‐ ‐ ‐ ‐ ‐ ‐ ‐ ‐ ‐ ‐ ‐ ‐|		
Ranked ≤10	896	492	78	945	791	473
Ranked ≤100	987	817	333	989	931	700
Median rank	1.5	16	237	1	2	14
Neutral mode						
Ranked #1	329			377		
Ranked #1–2	|‐ ‐ ‐ ‐ ‐ ‐ 37 ‐ ‐ ‐ ‐ ‐ ‐|			|‐ ‐ ‐ ‐ ‐ ‐ 139 ‐ ‐ ‐ ‐ ‐ ‐|		
Ranked #1–3	|‐ ‐ ‐ ‐ ‐ ‐ ‐ ‐ ‐ ‐ ‐ ‐ ‐ 1 ‐ ‐ ‐ ‐ ‐ ‐ ‐ ‐ ‐ ‐ ‐ ‐ ‐|			|‐ ‐ ‐ ‐ ‐ ‐ ‐ ‐ ‐ ‐ ‐ ‐ ‐ 40 ‐ ‐ ‐ ‐ ‐ ‐ ‐ ‐ ‐ ‐ ‐ ‐ ‐|		
Ranked ≤10	632	173	11	620	343	128
Ranked ≤100	779	366	64	822	597	312
Median rank	3	188.75	10,000	3	43.5	424.5

For all tests: uncaptured heterogeneity *u* = 0.5; captured heterogeneity split equally between the three genes (*p*_1_ = *p*_2_ = *p*_3_). Intersection filtering = results obtained using ranking based on intersection filtering; HetRank = results obtained using HetRank approach with interaction data from PINAmin2 network; Gene 1 = highest‐ranked of three disease genes in results; Gene 2 = second‐highest ranked; Gene 3 = lowest ranked. (a) Results using high‐coverage disease subnetworks (identified in PINAmin2 network). (b) Results using low‐coverage disease subnetworks (identified in PINA network).

When disease‐causing variants were assigned to OMIM disease subnetworks identified in PINAmin2, corresponding to high network coverage, our approach after network information was incorporated showed a consistent improvement in all modes compared to the ranking achieved using simple intersection filtering (Table 2a). We observed increases in the number of tests in which disease genes achieve high rankings (including all three genes being ranked in the top three places), as well as improved median ranks. In subsequent tests, we considered ranking in the top 10 genes as a successful test as we believe 10 prioritized genes to be a reasonable number for a researcher to study further in practice. The number of tests in which all three disease genes were ranked in the top 10 increased from 16 to 214 when our network‐informed approach was used in AD mode, from 82 to 733 in AR mode, and from 20 to 236 in neutral mode.

Of note, both HetRank and intersection filtering are slightly more effective in neutral mode than AD mode, despite being tested on the same simulated data designed to model an AD rare disease. This is most likely due to the fact that there is a small probability of homozygous variants being spiked into the simulated data (which are recognized by HetRank and intersection filtering in neutral mode but not AD mode). Nonetheless, the performance benefits of recognizing these spiked variants outweigh the costs of also recognizing additional homozygous background variation.

Table 2b shows the results obtained when disease‐causing variants were assigned to OMIM disease subnetworks with lower network coverage, this time identified in PINA (in this case, interactions used to model genetic heterogeneity may not be covered by the PINAmin2 network we use for ranking). Although the performance is slightly reduced, our approach still broadly improved the number of tests in which disease genes are ranked in the top positions compared with intersection filtering. In AD and neutral modes, the number of tests in which any disease gene ranks in the top 10 fell slightly, from 601 to 595 (AD) and from 632 to 620 (neutral). However, the number in which all three disease genes rank in the top 10 still showed substantial improvement, from 5 to 115 (AD) and from 11 to 128 (neutral). We therefore conclude that although the power to recover the top‐ranked disease gene may be slightly reduced, our approach can clearly boost the power to recover multiple genes involved in the disease process.

### Network Information Becomes More Beneficial With Increased Heterogeneity

Having demonstrated that our new approach can be a valuable additional analysis tool in exome sequencing studies we examined its performance in the presence of different levels of genetic heterogeneity. In each scenario and for each mode of operation (AD, AR, or neutral) we simulated 1,000 exome sequencing studies using randomly selected OMIM disease subnetworks of a fixed size (two, three, or four genes) to simulate captured heterogeneity, and a fixed level of uncaptured heterogeneity, *u* (20%, 40%, 60%, or 80%). Further, the captured heterogeneity could be balanced (each gene in the disease subnetwork being equally likely to be spiked into each exome) or unbalanced (one gene in the disease subnetwork being more likely to be spiked in than the others) giving a total of 24 scenarios per mode. As before, AD and neutral modes were tested with data that simulated an AD mode of inheritance, whereas AR mode was tested with data simulating an AR mode of inheritance.

All tests were performed using the high‐confidence PINAmin2 interaction network to inform gene rankings, using low coverage disease subnetworks (those identified in the PINA network meaning that some interactions may not be covered by PINAmin2). We measure the performance of our network‐informed HetRank approach by its improved ability to assign a rank of 10 or less to disease subnetwork genes relative to a ranking based on simple intersection filtering. Performance is also compared against the results achieved by HetRank without incorporating network information (i.e., omitting phase 3 in Fig. 1), and against the results achieved by BioGranat‐IG. As BioGranat‐IG will return all optimal subnetworks detected the user cannot specify the number of genes that will be returned. Therefore, a spiked gene was considered successfully prioritized if it was in the list of genes returned by BioGranat‐IG and the total number of genes returned by BioGranat‐IG was 10 or fewer.

**Figure 2:**
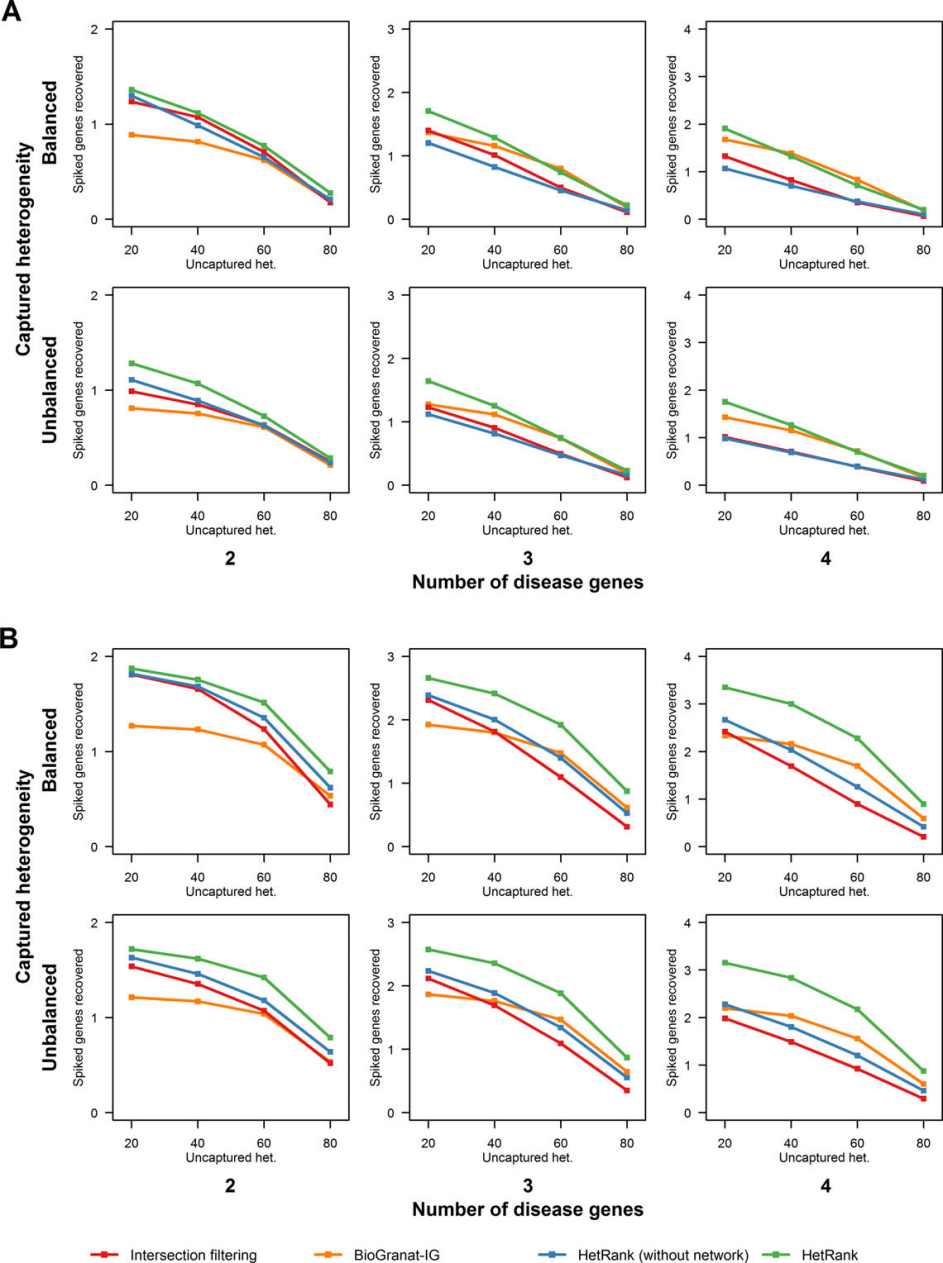
Performance of our approach at varying levels of genetic heterogeneity. Plots show the average number of spiked disease genes that could be prioritized (assigned a rank of 10 or less) across 1,000 simulated exome sequencing studies by the four methods tested (HetRank, HetRank excluding the network‐based step, BioGranat‐IG and simple intersection filtering). Different genetic heterogeneity scenarios are represented by the columns (number of genes in disease subnetworks modeling genetic heterogeneity), rows (whether this captured heterogeneity is balanced or unbalanced across disease subnetwork genes), and plot *x*‐axes (degree of genetic heterogeneity not captured by disease subnetwork genes). Results are also tabulated in Supp. Table S4. (a) Autosomal dominant mode results. (b) Autosomal recessive mode results.

Figures 2A and B present the results of these tests for the 24 scenarios in AD mode and AR mode, respectively. No substantial difference was detected between HetRank tests in AD and neutral mode; because of their material similarity to the AD mode results, the neutral mode results are shown in Supp. Figure S5. Indicators of performance for all scenarios are summarized in Supp. Tables S4 and S5.

In AD mode, network‐informed HetRank outperformed intersection filtering in all scenarios (Fig. 2A). Most of this improvement resulted from the addition of network information: when the network step was omitted, HetRank performed similarly to intersection filtering (in some cases being slightly outperformed, notably when captured heterogeneity is balanced across three‐ or four‐gene subnetworks). HetRank's performance also compared favorably against BioGranat‐IG, which struggles particularly at low levels of heterogeneity (for example, when captured heterogeneity is modeled by two genes and uncaptured heterogeneity is 40% or less). In such instances, each disease gene has sufficient signal that the other methods can perform reasonably well, even in the absence of network information. Conversely, BioGranat‐IG is unable to prioritize any genes not present or connected in the PINAmin2 input network (recall that captured heterogeneity is modeled by genes from PINA). At higher levels of heterogeneity, BioGranat‐IG performs better, indeed outperforming HetRank (although not substantially) in a few scenarios.

HetRank was also relatively robust to whether or not heterogeneity is balanced across disease genes compared to the other methods. Unbalanced heterogeneity leads to more signal for one of the genes in the disease subnetwork (making it theoretically easier to prioritize) at the expense of the others (making these harder to find). In nine of the 12 scenarios, there was a smaller drop (or larger increase, which tends to happen at higher levels of heterogeneity) relative to intersection filtering when comparing the number of disease genes found in unbalanced versus balanced captured heterogeneity scenarios. We also noted that HetRank was outperformed by BioGranat‐IG in fewer scenarios when captured heterogeneity is unbalanced than when it is balanced.

In AR mode, all methods performed better than in AD mode (Fig. 2B) because of the lower number of plausible AR disease‐causing variants in a typical exome (Supp. Table S2). However, HetRank showed a substantial advantage relative to the other methods, particularly at higher levels of heterogeneity (captured heterogeneity modeled by three or four disease genes). Relative to intersection filtering, most of the improved performance was again due to the incorporation of network information; however, HetRank also outperformed intersection filtering in all scenarios even when network information was not used. Further, network‐informed HetRank also performed better than BioGranat‐IG across all scenarios, suggesting that the variant‐ranking framework employed by HetRank can be particularly effective in the study of rare AR disease.

### HetRank Prioritizes Disease Genes in a Real Exome Sequencing Study

Finally, we tested HetRank's ability to prioritize the genes responsible for AOS using whole exome sequence data from 13 probands. It is known in advance that novel heterozygous truncating variants in *NOTCH1* cause AOS in two cases [Southgate et al., 2015] and a third harbors a novel heterozygous non‐synonymous variant in *DLL4* [Meester et al., 2015]. Dll4 is a member of the Delta family of Notch ligands [Shutter et al., 2000], and indeed the two genes are directly connected in the PINA network. However, *DLL4* is not present in the PINAmin2 network because of a lack of additional independent publications supporting interactions.

When PINAmin2 was used as the input network, HetRank assigned *NOTCH1* a rank of 3, demonstrating its ability to prioritize the true causal gene in the top three places. However, with just a single novel missense variant in one case exome (and in one control), and having no interactions in the network, *DLL4* was harder to prioritize and was ranked 1,047th (Supp. Table S6).

Using PINA as the input network, the rank of *NOTCH1* fell to 12. Although it achieved the same final score as when PINAmin2 was used (its relatively high degree of 65 in PINA prevents *NOTCH1* benefitting from evidence in neighboring genes), the additional interactions in the PINA network caused other genes to have their scores boosted ahead of *NOTCH1*. Conversely, the direct connection between *DLL4* and *NOTCH1* in PINA substantially improved the *DLL4* rank to (joint) 187th. The extent of genetic heterogeneity makes it challenging to prioritize *DLL4*: a single missense variant in one out of 13 case exomes provides a very limited evidence base. However, we subsequently showed that if just two additional case exomes carried a similar variant in *DLL4* then it would be prioritized in the top 10 by HetRank (Supp. Results and Supp. Table S7).

## Discussion

Genetic heterogeneity reduces the power of exome‐sequencing studies to identify the molecular basis of a monogenic disease because it limits the expected overlap of genes carrying deleterious mutations in unrelated affected individuals. This heterogeneity presents a challenging problem but in this study, we have shown that we can improve the prioritization of disease genes by incorporating information from an interaction network in a hypothesis‐free manner; that is, without specifying a set of candidate or “seed” genes.

Such an approach is particularly valuable when there is a high level of heterogeneity. There are currently many known sets of two to three interacting and disease‐causing genes (Table 1), but we expect to see larger connected sets in future as new disease‐causing genes are identified and as more comprehensive interaction networks are developed [Yu et al., 2011]. Gilissen et al. (2011) suggest that there is a scale of genetic heterogeneity broadly corresponding to disease prevalence, and our results support the use of HetRank in sequencing studies at the higher end of this scale. This could include studies of groups of patients with the same or very similar clinical phenotypes having unknown and potentially diverse molecular causes. A recent study of AR hereditary spastic paraplegias, for example, proposed eighteen novel candidate genes where a single variant in each was thought to be disease‐causing in different families [Novarino et al., 2014].

Our approach performs well in the presence of heterogeneity not captured by an interaction network (which we tested using the parameter *u*). This might include missing interaction data, disease variants that are not protein‐coding (intronic or intergenic variants), as well as potential non‐genetic disease causes such as epigenetic or environmental causes. As might be expected, though, Table 2 shows that better network coverage of interactions between disease‐causing genes improves HetRank results, as we also demonstrated when we applied HetRank to AOS data. Our approach does not specifically require a protein interaction network be used and as such it may be beneficial to seek increased coverage of the interactome by using networks which integrate different types of gene relationships [Lee et al., 2011; Vidal et al., 2011; Khurana et al., 2013].

Network information will not improve performance in every exome sequencing study. Even at higher levels of genetic heterogeneity there were examples in our simulated data where more disease genes are ranked in the top 10 using the simple intersection filtering method. This demonstrates what we already know: it is important that a researcher also considers the evidence for a gene's involvement in a disease independently of the genes with which it interacts. One way to do this could be to consider HetRank gene prioritizations alongside those obtained by intersection filtering when analyzing exome sequencing results.

On a similar note, users of HetRank should understand the limitations of the tool. At higher levels of genetic heterogeneity, we saw many simulated studies in which no disease genes could be ranked in the top 10 and when applied to real AOS exomes HetRank could prioritize only one of the two causal genes. Furthermore, for any given monogenic disease, the model underlying HetRank (that genetic heterogeneity can be at least partially explained by interacting genes) may be inappropriate; the tool itself cannot determine if this is the case. Even for an exome sequencing study in which disease‐causing genes are ranked in the top 10 by HetRank (our measure of success in performance tests), those 10 will also include non‐causal genes. A thorough examination of the top‐ranked genes is likely to be required in order to pick out the genes of interest. However, HetRank is able to provide the user with a starting point; by studying the high‐ranking genes and the variants contained within those and their interaction partners, and combining this with existing functional annotation or disease‐specific knowledge, hypotheses can emerge concerning putative disease mechanisms which can be taken forward for further testing.

An important point to note is that the use of the HetRank framework does not preclude the use of other tools designed for gene or variant prioritization, but can be considered complementary to existing approaches. Variant effect prediction tools such as SIFT [Kumar et al., 2009] and PolyPhen [Adzhubei et al., 2010] can be incorporated into HetRank by including their prediction scores as variant‐ranking criteria, and nothing prevents the same approach being taken for existing tools such as eXtasy [Sifrim et al., 2013], Exomiser [Robinson et al., 2014], or CADD [Kircher et al., 2014], which themselves integrate diverse sources of evidence for deleteriousness or disease involvement. Even if a user relies entirely on an existing tool to integrate evidence sources, HetRank can still make a valuable contribution to addressing genetic heterogeneity by adjusting this evidence with reference to an interaction network.

The assumption that proximal genes in an interaction network can account for similar biological phenomena in different individuals suggests that implicit functional pathways underlie network structure. HetRank seeks to benefit from such pathways through the joint analysis of several affected individuals, for each of whom only one of the pathway genes may be implicated. Similar approaches have previously been suggested in related fields, such as gene expression [Ulitsky et al., 2010] and cancer genomics [Vandin et al., 2011]. Our results demonstrate that with methods adapted to the particular challenges of whole exome sequence analysis, interaction networks can also be profitably exploited in this way when the biological phenomenon of interest is a rare monogenic disease.

In summary, we have presented HetRank, a flexible analysis method which uses variant‐ranking in unrelated exomes to combine several sources of evidence for involvement in a monogenic disease. In an interaction network, neighboring genes that score highly in different affected individuals improve the evidence for one another's involvement in the disease process. The final prioritization is obtained by combining adjusted gene scores across all exomes in the study, and we have demonstrated that this can effectively deal with a considerable degree of genetic heterogeneity.

*Disclosure statement*: The authors declare no conflict of interest.

## Supporting information

Figure S1 – Examples of variant score profiles
Figure S2 – Method for simulation of exome sequencing studies
Figure S3 – Distributions of variant annotations
Figure S4 – Interactions between genes involved in the same disease occur frequently
Figure S5 – Performance of HetRank in neutral mode at varying levels of genetic heterogeneity
Table S1 – OMIM Disease Subnetworks
Table S2 – Performance of intersection filtering method using various parameters
Table S3 – Performance of BioGranat‐IG method using various parameters
Table S4 – Ability of all methods to recover disease subnetworks under varying levels of genetic heterogeneity
Table S5 – Improved ability to recover disease subnetworks under varying levels of genetic heterogeneity using network‐informed HetRank approach relative to simple intersection filtering
Table S6 – Summary of top prioritised genes in 13 Adams‐Oliver syndrome exomes
Table S7 – Ability to prioritise Adams‐Oliver syndrome genes with additional disease‐causing variants
