# Supplementary material for: Network‐Informed Gene Ranking Tackles Genetic Heterogeneity in Exome‐Sequencing Studies of Monogenic Disease
Source: Hum Mutat. 2015 Oct 7;36(12):1135–44. doi: 10.1002/humu.22906 (PMC4982032; doi:10.1002/humu.22906)
Supplement: Supplementary file 1 — Figure S1 – Examples of variant score profiles Figure S2 – Method for simulation of exome sequencing studies Figure S3 – Distributions of variant annotations Figure S4 – Interactions between genes involved in the same disease occur frequently Figure S5 – Performance of HetRank in neutral mode at varying levels of genetic heterogeneity Table S1 – OMIM Disease Subnetworks Table S2 – Performance of intersection filtering method using various parameters Table S3 – Performance of BioGranat‐IG method using various parameters Table S4 – Ability of all methods to recover disease subnetworks under varying levels of genetic heterogeneity Table S5 – Improved ability to recover disease subnetworks under varying levels of genetic heterogeneity using network‐informed HetRank approach relative to simple intersection filtering Table S6 – Summary of top prioritised genes in 13 Adams‐Oliver syndrome exomes Table S7 – Ability to prioritise Adams‐Oliver syndrome genes with additional disease‐causing variants [file HUMU-36-1135-s001.pdf]

Supporting Information for the article:

Network-informed gene ranking tackles genetic heterogeneity in exome-sequencing studies of monogenic disease

Nick Dand, Reiner Schulz, Michael E. Weale, Laura Southgate, Rebecca J. Oakey, Michael A. Simpson and Thomas Schlitt

Supplementary Methods

Degree constrained network-permutation approach

We hypothesised that the number of OMIM disease subnetworks identified in the PINA and PINAmin2 networks was not a simple consequence of the number of disease genes in each network. To test this we checked for disease subnetworks in 10,000 random permutations of each network.

However, if disease genes are more highly connected than average in the network (for example, due to being relatively well-studied) this could bias the test, with disease genes more likely to be connected in the real network than the permuted network if node degree is not accounted for. Therefore we generated permuted networks using a degree-constrained node-shuffling approach, which has been previously described [Lehne, 2012] and applied [Prescott, et al., 2015].

Briefly, node labels are preferentially swapped with nodes of similar degree. For node (i.e. gene) *g* this is achieved by listing all other network nodes in increasing order of degree difference relative to *g* (with nodes of equal degree ordered uniformly randomly); a one-tailed normal distribution centred at the top of this list is then used to select a node and labels are swapped. This is repeated for all network nodes. The default standard deviation of 5.0 nodes was used.

Identification of optimal parameters for intersection filtering

By seeking the filtering thresholds which give the best possible performance for the intersection filtering method, we will obtain a fair assessment of the extent to which HetRank can improve on this method. We therefore tested a range of intersection filtering criteria to identify the optimal combination of parameters.

Testing was performed using 1,000 simulated exome sequencing studies, as described in the main text. Genetic heterogeneity was modelled by disease networks of three genes, with each gene equally likely to be disease-causing ($p_1 = p_2 = p_3$). Heterogeneity not captured by the disease subnetwork was set at 50% ($u = 0.5$). Disease networks generated using both PINA and PINAmin2 networks were tested. Each study comprised 20 simulated case exomes, including spiked disease mutations, and 180 exomes acting as healthy controls.

Filtering criteria tested were:

- variant effect
    - include truncating variants only, or
    - include truncating and protein-altering variants
- 1000 Genomes and EVS alternative allele frequency
    - include novel variants only (both frequencies = 0), or
    - include novel and rare variants (both frequencies ≤ 0.001)
- presence of similar variants in control exomes
    - do not filter genes relative to controls, or
    - exclude genes in which 5+ of control exomes carry variant(s) with consistent mode of inheritance after filtering on frequency and variant effect, or
    - exclude genes in which 10+ of control exomes carry such variants.

Results of these tests are presented in Supp. Table S2. For each mode (autosomal dominant, autosomal recessive and neutral), the best results are in the cells highlighted in green.

Based on their ability to rank the highest number of spiked disease genes in the top ten, the two best-performing combinations of filters are for novel altering variants when genes observed in ten or more controls are excluded and for rare altering variants when genes observed in five or more controls are excluded. However, the latter combination was preferred because it consistently ranked more spiked disease genes in the top three and because it was never substantially outperformed by the former when assessing the genes ranked in the top ten. Therefore all intersection filtering results presented in the main text are based on **rare altering variants with genes observed in five or more controls being excluded**.

Identification of optimal parameters for BioGranat-IG

As with the intersection filtering method, we aim to identify the BioGranat-IG parameters which give the best possible performance in order to make a fair comparison against HetRank.

Testing was performed on the same set of simulated data as described in the previous section (that is, based on disease subnetworks of three genes with balanced captured heterogeneity and 50% uncaptured heterogeneity). Since BioGranat-IG requires filtered gene lists, we used the optimal filtering procedure determined in the previous section (we filter out synonymous variants, variants with frequency > 0.001 and genes with post-filtering variants in five or more control exomes).

The input network for BioGranat-IG was PINAmin2 or was derived from PINAmin2 (see below), and we tested both high-coverage disease subnetworks (drawn from PINAmin2) and low-coverage disease subnetworks (drawn from PINA; interactions used to model genetic heterogeneity may not be present in PINAmin2).

Parameters tested were:

- BioGranat-IG search algorithm [see Dand, et al., 2013]
    - using triplet search (with default settings: results flexibility parameters = 0), or
    - using minimum and multi-minimum distance heuristic search (with default settings: results flexibility parameters = 0, searches limited to ten genes, 1,000 iterations per gene and 2,000,000 iterations total)
- treatment of input network hub genes (hub genes tend to be over-represented in BioGranat-IG results and it is advisable to use an input network with highly-connected hub genes removed)
    - use full PINAmin2 network, or
    - use PINAmin2_d50, a network constructed by removing nodes of degree 50 or more from PINAmin2.

Results of these tests are presented in Supp. Table S3. For each mode (autosomal dominant, autosomal recessive and neutral), the best results are in the cells highlighted in green. Note that BioGranat-IG's exact triplet search was unable to produce results for comparison in the full PINAmin2 network due to the network's complexity.

The results show that when considering the average number of spiked disease genes that are ranked in the top ten (that is, by being returned in a list of ten genes or fewer), BioGranat-IG is most effective across all scenarios when its **triplet search algorithm is employed in the hub-free version of the network, PINAmin2_d50**. Therefore all BioGranat-IG results presented in the main text are based on this combination of parameters.

Supplementary Results

HetRank would better prioritise Adams-Oliver syndrome genes with variants in additional exomes

HetRank was able to rank *NOTCH1* (novel truncating variants in 2 exomes) in 12th position and *DLL4* (novel protein-altering variant in 1 exome) in (joint) 187th position when applied to 13 AOS exomes using the PINA network.

We tested whether additional variants in these genes would improve their rankings. To do this we added additional copies of the known causal variants to 1 or more of the 10 AOS exomes not harbouring *NOTCH1* or *DLL4* mutations (for which the genetic cause remains unknown). Results are summarised in Supp. Table S7.

When the causal (protein-altering) *DLL4* variant is added to 2 additional exomes, *DLL4* is ranked seventh by HetRank. Due to its relatively high degree of 65 in the PINA network, *NOTCH1* does not benefit from the increased evidence in its direct neighbour DLL4, and it is ranked 13th (dropping one place to accommodate *DLL4* ahead of it).

When instead one of the causal (truncating) *NOTCH1* variants is added to 1 additional exome, *NOTCH1* is ranked first by HetRank. *DLL4* has a relatively low degree of 3 in the network and does benefit from evidence in its direct neighbour *NOTCH1*, so that its rank is improved to 47. As causal *NOTCH1* variants are added to further exomes, *NOTCH1* remains the top-ranked gene and *DLL4*'s rank continues to improve. By the time 4 additional exomes have *NOTCH1* variants, the rank of *DLL4* has peaked at 20. This is because all of the genes ranked 2-19 are also direct neighbours of *NOTCH1* in the PINA network so that they will all benefit from each subsequent exome that receives a *NOTCH1* variant.

Supplementary References

Dand N, Sprengel F, Ahlers V, Schlitt T. 2013. BioGranat-IG: a network analysis tool to suggest mechanisms of genetic heterogeneity from exome-sequencing data. Bioinformatics 29:733-741.

Lehne B. 2012. Computational Analyses of Complex Diseases at the Gene and Network Levels [Doctoral Thesis]: King's College London, UK.

Prescott NJ, Lehne B, Stone K, Lee JC, Taylor K, Knight J, Papouli E, Mirza MM, Simpson MA, Spain SL, Lu G, Fraternali F et al. 2015. Pooled sequencing of 531 genes in inflammatory bowel disease identifies an associated rare variant in BTNL2 and implicates other immune related genes. PLoS Genet 11:e1004955.

Figure S1 – Examples of variant score profiles

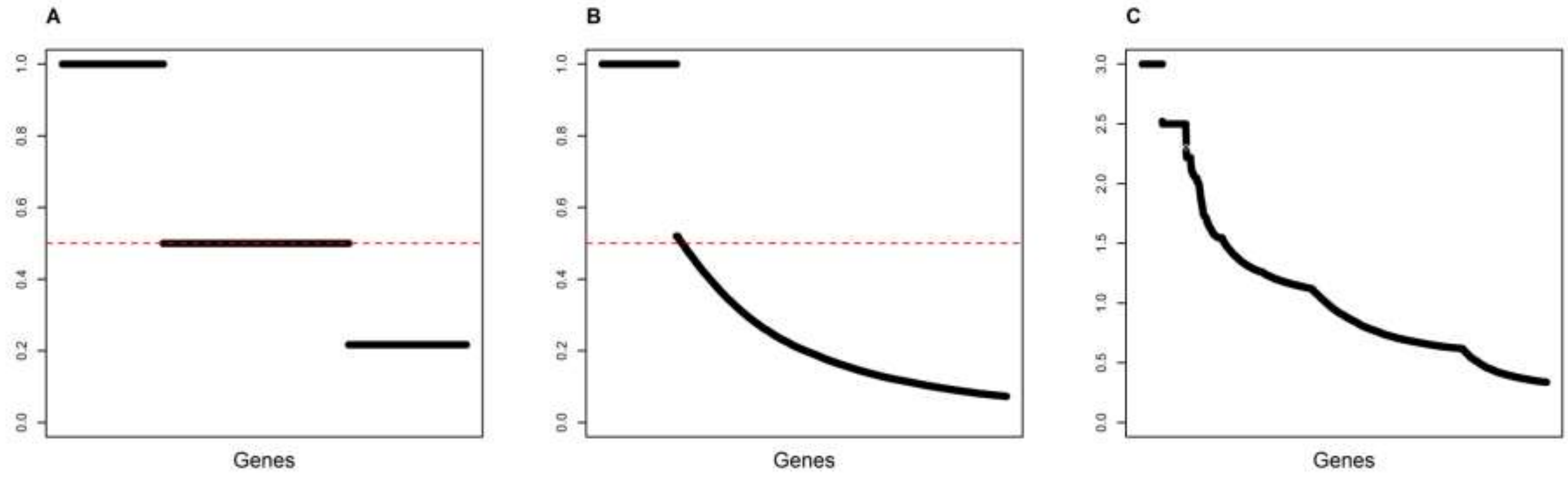

Plots show example gene scores in a single exome based on different variant annotations (with gene scores derived from variant scores in neutral mode). Gene scores are plotted in decreasing order. **(a)** Gene scores based on variant effect only, ranked according to "truncating > altering > synonymous" and with a filter-equivalent threshold value set at "altering". This results in truncating variants all receiving a score of 1, while altering variants are forced to receive a score of 0.5 (red dotted line). Scaling of variant scores with respect to filter-equivalent threshold values works as follows. Let y be the reciprocal of the rank assigned to variant *v* with respect to the annotation (where average rank is used to resolve ties). Then the scaled variant score y^* is set equal to $0.5 + 0.5 \times (y - y_{thresh})/(y_{max} - y_{thresh})$ if $y \geq y_{thresh}$ or $0.5 \times y/y_{thresh}$ if $y < y_{thresh}$ (where y_{thresh} is the reciprocal rank of the variant whose annotation value is at or is closest inside the filter-equivalent threshold value, and y_{max} is the largest reciprocal rank among all variants – which is equal to 1 if there is a unique highest-ranking variant but < 1 if there are multiple joint-top-ranked variants). **(b)** Gene scores based on EVS alternative allele frequency only, ranked in increasing order and with a filter-equivalent threshold value set at 0.001. This results in novel variants all receiving a score of 1; gene scores cross the red dotted line (score of 0.5) at a gene whose rarest variant has frequency closest to 0.001. Genes scores based on 1000 Genomes alternative allele frequency only give a similar plot. **(c)** Gene scores based on all three annotations (variant effect, EVS alternative allele frequency and 1000 Genomes alternative allele frequency) together with order and filter-equivalent threshold values as previously.

Figure S2 – Method for simulation of exome sequencing studies

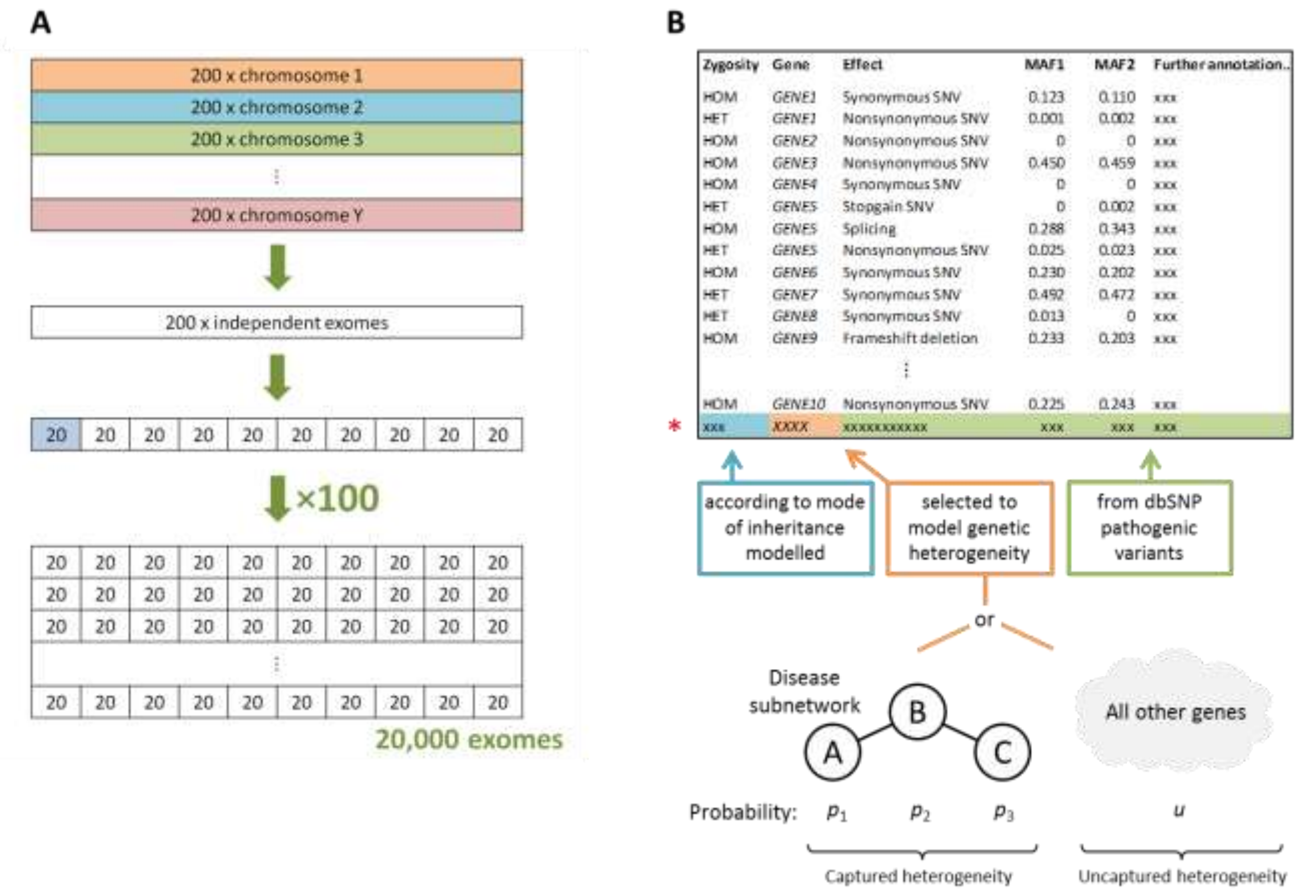

(a) Unspiked exomes are generated in blocks of 200 through random selection (without replacement) of variants by chromosome from 388 sequenced exomes. Each block of 200 unspiked exomes can be split into ten "studies" of 20 exomes each. For each study the other 180 exomes in the block share no genetic material and can therefore be used as unaffected control exomes in their unspiked state. **(b)** To simulate rare disease exome sequencing studies, each of the 20 annotated exomes in a study are "spiked" with an additional disease-causing variant (red asterisk), or two variants in the event that a compound heterozygous mutation is modelled. Annotation for the spiked variant(s) are determined as follows: zygosity is chosen to model specific modes of disease inheritance; gene name is randomly selected to model genetic heterogeneity ("captured" heterogeneity from a disease subnetwork with probability 1–u or "uncaptured" with probability u); all other annotation is randomly selected from a set of pathogenic variants from dbSNP. When captured heterogeneity is generated from a disease subnetwork of three genes (genes *A*, *B* and *C* here) the probabilities that each gene is picked are denoted p_1, p_2 and p_3 (such that $p_1 + p_2 + p_3 + u = 1$)

Figure S3 – Distributions of variant annotations

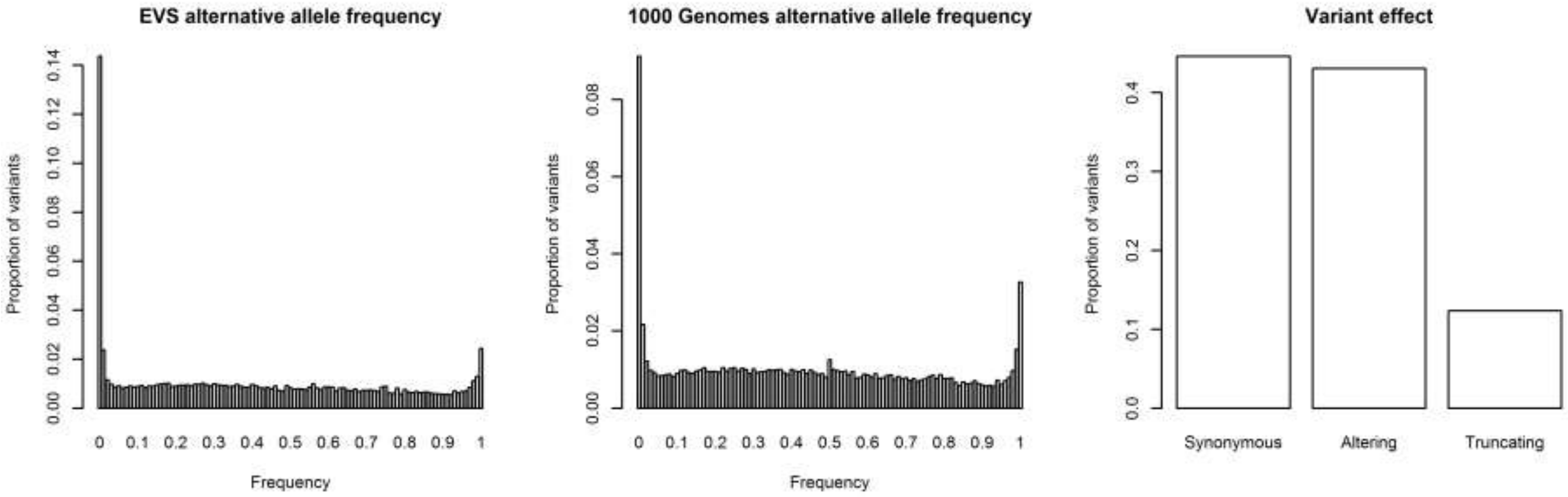

Plots show distributions for the three variant annotations used (EVS alternative allele frequency, 1000 Genomes alternative allele frequency and variant effect) across all 20,000 (unspiked) exomes, generated as described in the main text.

.

Figure S4 – Interactions between genes involved in the same disease occur frequently

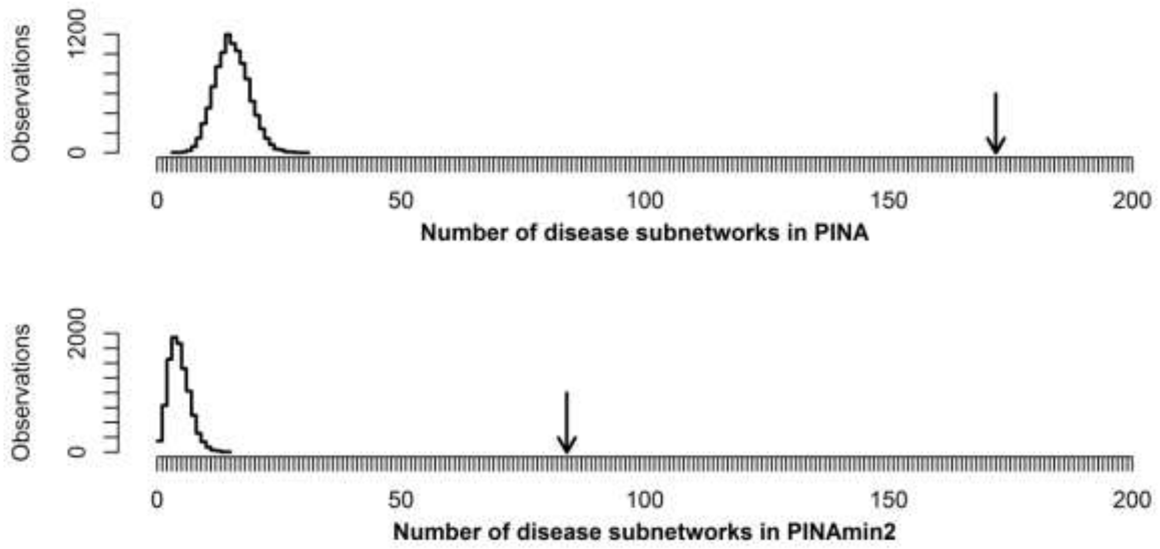

Frequency plots showing the number of disease subnetworks (connected sets of two or more genes causing the same disease) identified in 10,000 random degree-constrained permutations of the PINA and PINAmin2 networks. Arrows indicate number of disease networks found in the original networks.

Figure S5 – Performance of HetRank in neutral mode at varying levels of genetic heterogeneity

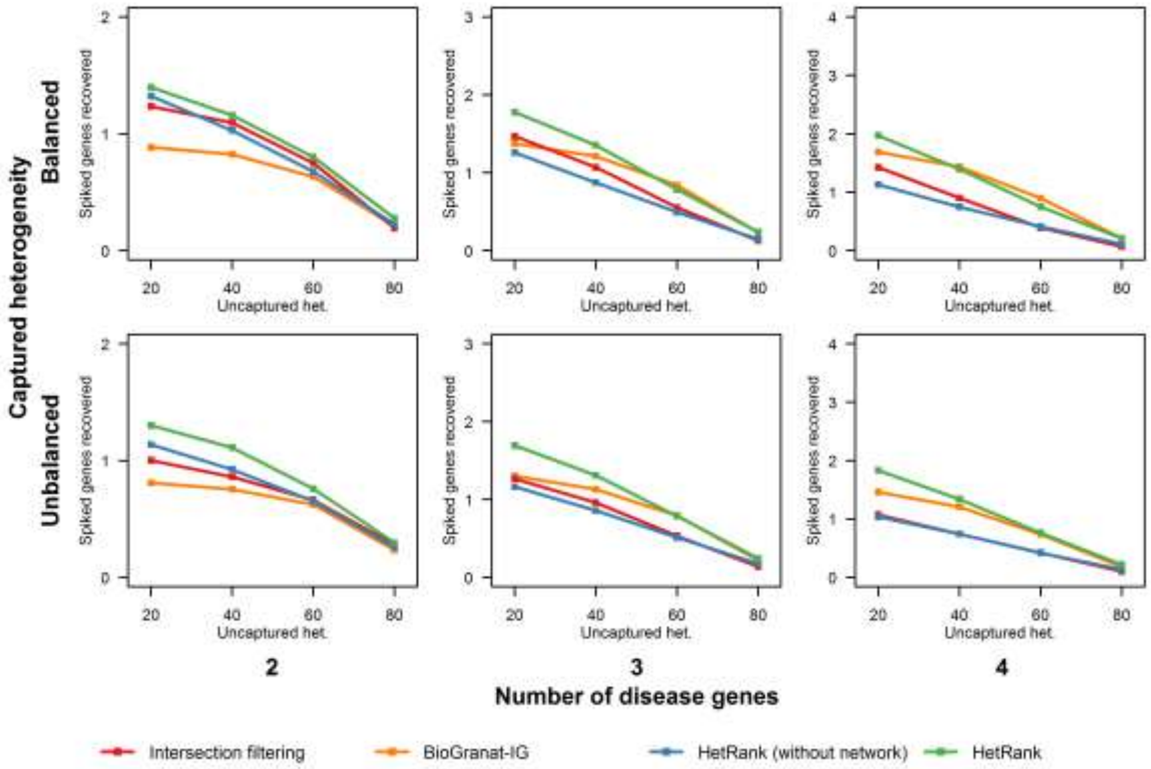

Results for HetRank's neutral mode; corresponding plots for Autosomal Dominant and Autosomal Recessive modes are given in main text (Figure 2). Plots show the average number of spiked disease genes that could be prioritised (assigned a rank of ten or less) across 1,000 simulated exome sequencing studies by the four methods tested (HetRank, HetRank excluding the network-based step, BioGranat-IG and simple intersection filtering). Different genetic heterogeneity scenarios are represented by the columns (number of genes in disease subnetworks modelling genetic heterogeneity), rows (whether this captured heterogeneity is balanced or unbalanced across disease subnetwork genes), and plot x-axes (degree of genetic heterogeneity not captured by disease subnetwork genes). Results are also tabulated in Supp. Table S4.

Table S1 – OMIM Disease Subnetworks

Network	Generalised disease term	Causal genes connected in network
PINA	3-M syndrome	*CUL7, OBSL1*
PINAmin2	3-M syndrome	*CUL7, OBSL1*
PINA	46XY sex reversal	*MAP3K1, SRY*
PINA	Acne inversa familial	*PSEN1, NCSTN, PSENEN*
PINAmin2	Acne inversa familial	*PSEN1, NCSTN, PSENEN*
PINA	Afibrinogenemia congenital	*FGB, FGA*
PINAmin2	Afibrinogenemia congenital	*FGA, FGB*
PINA	Agammaglobulinemia	*CD79A, CD79B, BLNK, IGHM*
PINAmin2	Agammaglobulinemia	*CD79A, BLNK, CD79B*
PINA	Albinism oculocutaneous type	*TYR, TYRP1*
PINAmin2	Albinism oculocutaneous type	*TYR, TYRP1*
PINA	Arrhythmogenic right ventricular dysplasia	*JUP, DSP, DSC2, DSC3, DSG2, PKP2*
PINAmin2	Arrhythmogenic right ventricular dysplasia	*JUP, DSP*
PINA	Arthrogryposis renal dysfunction and cholestasis	*VPS33B, VIPAS39*
PINAmin2	Arthrogryposis renal dysfunction and cholestasis	*VPS33B, VIPAS39*
PINA	Atrial fibrillation familial	*KCNQ1, KCNE2*
PINA	Axenfeld-Rieger syndrome type	*FOXC1, PITX2*
PINA	Baraitser-Winter syndrome	*ACTB, ACTG1*
PINAmin2	Baraitser-Winter syndrome	*ACTB, ACTG1*
PINA	Bardet-Biedl syndrome	*ARL6, BBS1, BBS4, BBS12, BBS7, BBS9, BBS10, BBS2, MKKS, BBS5, TTC8*
PINAmin2	Bardet-Biedl syndrome	*BBS1, BBS4, BBS7, BBS9, BBS12, BBS2, ARL6, MKKS, BBS5, TTC8*
PINA	Bare lymphocyte syndrome type	*TAP1, TAPBP*
PINAmin2	Bare lymphocyte syndrome type	*TAPBP, TAP1*
PINA	Bare lymphocyte syndrome type complementation group	*RFX5, RFXAP*
PINA	Basal cell carcinoma somatic	*PTCH1, SMO, PTCH2*
PINA	Bernard-Soulier syndrome type	*GP9, GP1BB*
PINAmin2	Bernard-Soulier syndrome type	*GP1BB, GP9*
PINA	Brachydactyly type	*BMPR1B, BMP2, GDF5, NOG*
PINAmin2	Brachydactyly type	*BMP2, BMPR1B, GDF5, NOG*
PINA	Bradyopsia	*RGS9, RGS9BP*
PINA	Breast cancer	*TP53, ESR1, PPM1D*
PINA	Bronchiectasis with or without elevated sweat chloride	*SCNN1B, SCNN1A, SCNN1G*
PINA	Brugada syndrome	*CACNA1C, CACNB2*
PINA	C1q deficiency	*C1QA, C1QB, C1QC*
PINAmin2	C1q deficiency	*C1QA, C1QB, C1QC*
PINA	C8 deficiency type	*C8A, C8B*
PINA	Cardiofaciocutaneous syndrome	*BRAF, MAP2K1, MAP2K2*
PINAmin2	Cardiofaciocutaneous syndrome	*BRAF, MAP2K2, MAP2K1*
PINA	Cardiomyopathy familial hypertrophic	*TNNI3, TPM1, TNNT2, TNNC1*

Network	Generalised disease term	Causal genes connected in network
PINA	Cardiomyopathy familial hypertrophic	*MYBPC3, TTN*
PINAmin2	Cardiomyopathy familial hypertrophic	*TNNI3, TNNT2, TNNC1*
PINA	Cataract Coppock-like	*CRYBB2, CRYGC*
PINA	Cerebrooculofacioskeletal syndrome	*ERCC2, ERCC6, ERCC5*
PINAmin2	Cerebrooculofacioskeletal syndrome	*ERCC5, ERCC6*
PINA	Charcot-Marie-Tooth disease axonal type	*HSPB1, HSPB8*
PINAmin2	Charcot-Marie-Tooth disease axonal type	*HSPB1, HSPB8*
PINA	Charcot-Marie-Tooth disease type	*NEFL, MTMR2*
PINA	Charcot-Marie-Tooth disease type	*PMP22, MPZ*
PINA	Choriodal dystrophy central areolar	*PRPH2, PRPH*
PINA	Cirrhosis cryptogenic	*KRT18, KRT8*
PINAmin2	Cirrhosis cryptogenic	*KRT8, KRT18*
PINA	Cockayne syndrome type	*ERCC6, ERCC8*
PINAmin2	Cockayne syndrome type	*ERCC8, ERCC6*
PINA	Colorectal cancer hereditary nonpolyposis type	*MLH1, MSH2, PMS2, MSH6*
PINAmin2	Colorectal cancer hereditary nonpolyposis type	*MLH1, MSH2, MSH6, PMS2*
PINA	Colorectal cancer somatic	*EP300, APC, CTNNB1, AKT1, BRAF, BUB1B, DLC1, AXIN2*
PINA	Colorectal cancer somatic	*NRAS, PIK3CA*
PINAmin2	Colorectal cancer somatic	*AKT1, APC, CTNNB1, EP300, AXIN2*
PINA	Combined cellular and humoral immune defects with granulomas	*RAG1, RAG2*
PINA	Cone-rod dystrophy	*GUCA1A, GUCY2D*
PINAmin2	Cone-rod dystrophy	*GUCA1A, GUCY2D*
PINA	Congenital disorder of glycosylation type	*DPM1, DPM2, DPM3*
PINA	Congenital disorder of glycosylation type	*COG1, COG4, COG7, COG5, COG6*
PINAmin2	Congenital disorder of glycosylation type	*COG1, COG4, COG7, COG6, COG5*
PINAmin2	Congenital disorder of glycosylation type	*DPM1, DPM3*
PINA	Cornelia de Lange syndrome	*RAD21, SMC3, NIPBL*
PINAmin2	Cornelia de Lange syndrome	*RAD21, SMC3*
PINA	Cowden syndrome	*AKT1, PTEN*
PINA	Cutis laxa autosomal recessive type	*EFEMP2, FBLN5*
PINA	Deafness autosomal dominant	*SIX1, EYA4*
PINA	Deafness autosomal recessive	*MYO7A, CDH23, DFNB31, MYO15A*
PINA	Dejerine-Sottas disease	*PMP22, MPZ*
PINA	Dementia Lewy body	*SNCA, SNCB*
PINA	Diabetes mellitus permanent neonatal	*INS, GCK*
PINA	Diabetes mellitus transient neonatal	*ABCC8, KCNJ11*
PINA	Diamond-Blackfan anemia	*RPL5, RPL11*
PINAmin2	Diamond-Blackfan anemia	*RPL5, RPL11*
PINA	Dysfibrinogenemia type	*FGB, FGG*
PINAmin2	Dysfibrinogenemia type	*FGB, FGG*
PINA	Ehlers-Danlos syndrome type	*COL1A1, COL1A2*
PINA	Ehlers-Danlos syndrome type	*COL5A2, COL5A1*
PINAmin2	Ehlers-Danlos syndrome type	*COL1A1, COL1A2*

Network	Generalised disease term	Causal genes connected in network
PINA	Epidermolysis bullosa simplex type	*KRT5, KRT14*
PINAmin2	Epidermolysis bullosa simplex type	*KRT14, KRT5*
PINA	Epidermolysis bullosa junctional type	*ITGB4, COL17A1*
PINA	Epidermolysis bullosa junctional type	*LAMB3, LAMA3, LAMC2*
PINAmin2	Epidermolysis bullosa junctional type	*LAMB3, LAMA3, LAMC2*
PINA	Epilepsy nocturnal frontal lobe	*CHRNB2, CHRNA4*
PINAmin2	Epilepsy nocturnal frontal lobe	*CHRNB2, CHRNA4*
PINA	Epilepsy progressive myoclonic 2B (Lafora)	*EPM2A, NHLRC1*
PINAmin2	Epilepsy progressive myoclonic 2B (Lafora)	*EPM2A, NHLRC1*
PINA	Epiphyseal dysplasia multiple	*COL9A1, COMP, MATN3*
PINA	Episodic ataxia type	*CACNA1A, CACNB4*
PINA	Erythrocytosis familial	*VHL, EPAS1, EGLN1*
PINAmin2	Erythrocytosis familial	*VHL, EPAS1, EGLN1*
PINA	Exostoses multiple type	*EXT1, EXT2*
PINA	Fanconi anemia complementation group	*FANCA, FANCG, FANCM, BRCA2, PALB2, FANCC, FANCB, FANCI, FANCD2, FANCE, FANCF, BRIP1*
PINAmin2	Fanconi anemia complementation group	*FANCG, BRCA2, FANCA, FANCM, FANCC, FANCD2, FANCE, PALB2, FANCF, FANCB, FANCI*
PINA	Foveomacular dystrophy adult-onset with choroidal neovascularization	*PRPH2, PRPH*
PINA	Frontonasal dysplasia	*ALX1, ALX4*
PINA	Glanzmann thrombasthenia	*ITGB3, ITGA2B*
PINAmin2	Glanzmann thrombasthenia	*ITGA2B, ITGB3*
PINA	Griscelli syndrome type	*RAB27A, MLPH, MYO5A*
PINAmin2	Griscelli syndrome type	*MLPH, RAB27A, MYO5A*
PINA	Hemangioma capillary infantile somatic	*KDR, FLT4*
PINAmin2	Hemangioma capillary infantile somatic	*KDR, FLT4*
PINA	Hemochromatosis type	*HAMP, SLC40A1*
PINA	Hemophagocytic lymphohistiocytosis familial	*STX11, FHL5*
PINA	Hepatocellular carcinoma somatic	*CTNNB1, AXIN1, CASP8*
PINAmin2	Hepatocellular carcinoma somatic	*AXIN1, CTNNB1*
PINA	Hermansky-Pudlak syndrome	*DTNBP1, BLOC1S3*
PINA	Hermansky-Pudlak syndrome	*HPS1, HPS4*
PINA	Hermansky-Pudlak syndrome	*HPS6, HPS5*
PINAmin2	Hermansky-Pudlak syndrome	*BLOC1S3, DTNBP1*
PINAmin2	Hermansky-Pudlak syndrome	*HPS6, HPS5*
PINA	Hyperinsulinemic hypoglycemia familial	*ABCC8, KCNJ11*
PINA	Hypogonadotropic hypogonadism with or without anosmia	*FGFR1, FGF8*
PINA	Hypogonadotropic hypogonadism with or without anosmia	*GNRH1, GNRHR*
PINA	Hypogonadotropic hypogonadism with or without anosmia	*TACR3, TAC3*
PINA	Hypogonadotropic hypogonadism with or without anosmia	*KISS1, KISS1R*

Network	Generalised disease term	Causal genes connected in network
PINA	Immune dysfunction with T-cell inactivation due to calcium entry defect	*ORAI1, STIM1*
PINAmin2	Immune dysfunction with T-cell inactivation due to calcium entry defect	*ORAI1, STIM1*
PINA	Immunodeficiency common variable	*CD19, CR2, CD81*
PINAmin2	Immunodeficiency common variable	*CD19, CR2, CD81*
PINA	Iridogoniodysgenesis type	*FOXC1, PITX2*
PINA	Kabuki syndrome	*KDM6A, MLL2*
PINAmin2	Kabuki syndrome	*KDM6A, MLL2*
PINA	LADD syndrome	*FGFR2, FGF10*
PINAmin2	LADD syndrome	*FGF10, FGFR2*
PINA	LEOPARD syndrome	*RAF1, BRAF*
PINAmin2	LEOPARD syndrome	*RAF1, BRAF*
PINA	Leigh syndrome due to mitochondrial complex I deficiency	*NDUFS3, NDUFA9*
PINAmin2	Leigh syndrome due to mitochondrial complex I deficiency	*NDUFS3, NDUFA9*
PINA	Leukemia acute myeloid	*CEBPA, RUNX1*
PINA	Leukemia acute promyelocytic type	*ZBTB16, PML*
PINA	Leukoencephalopathy with vanishing white matter	*EIF2B1, EIF2B2, EIF2B5, EIF2B3*
PINAmin2	Leukoencephalopathy with vanishing white matter	*EIF2B5, EIF2B1*
PINA	Li-Fraumeni syndrome	*CHEK2, TP53*
PINAmin2	Li-Fraumeni syndrome	*TP53, CHEK2*
PINA	Liddle syndrome	*SCNN1B, SCNN1G*
PINA	Lissencephaly	*TUBA1A, PAFAH1B1*
PINA	Loeys-Dietz syndrome type	*SMAD3, TGFBR1, TGFBR2, TGFB2*
PINAmin2	Loeys-Dietz syndrome type	*TGFBR1, SMAD3, TGFBR2, TGFB2*
PINA	MODY type	*HNF4A, HNF1A*
PINA	Macular dystrophy	*PRPH2, PRPH*
PINA	Macular dystrophy patterned	*PRPH2, PRPH*
PINA	Macular dystrophy vitelliform	*PRPH2, PRPH*
PINA	Maple syrup urine disease type	*BCKDHA, BCKDHB*
PINAmin2	Maple syrup urine disease type	*BCKDHA, BCKDHB*
PINA	Meckel syndrome	*TMEM67, MKS1*
PINA	Meier-Gorlin syndrome	*CDC6, CDT1, ORC4, ORC1, ORC6*
PINAmin2	Meier-Gorlin syndrome	*CDC6, ORC1, ORC4, CDT1, ORC6*
PINA	Mental retardation X-linked	*GDI1, FTSJ1*
PINA	Mental retardation autosomal dominant	*SMARCA4, ARID1A, CTNNB1, GRIN1, GRIN2B, ARID1B, SMARCB1, CDH15, SYNGAP1, CDH3, CACNG2*
PINAmin2	Mental retardation autosomal dominant	*SMARCA4, ARID1A, SMARCB1, ARID1B*
PINAmin2	Mental retardation autosomal dominant	*CTNNB1, CDH3*
PINAmin2	Mental retardation autosomal dominant	*GRIN2B, GRIN1*

Network	Generalised disease term	Causal genes connected in network
PINA	Methemoglobinemia type	*CYB5A, CYB5R3*
PINA	Microcephaly primary autosomal recessive	*CEP135, CENPJ*
PINA	Mismatch repair cancer syndrome	*MLH1, MSH2, PMS2, MSH6*
PINAmin2	Mismatch repair cancer syndrome	*MLH1, MSH2, MSH6, PMS2*
PINA	Mitochondrial complex I deficiency	*NDUFS3, NDUFS2*
PINAmin2	Mitochondrial complex I deficiency	*NDUFS3, NDUFS2*
PINA	Muir-Torre syndrome	*MLH1, MSH2*
PINAmin2	Muir-Torre syndrome	*MLH1, MSH2*
PINA	Multiple pterygium syndrome type	*CHRND, CHRNA1, CHRNG*
PINAmin2	Multiple pterygium syndrome type	*CHRNA1, CHRND*
PINA	Muscular dystrophy limb-girdle type	*DYSF, TCAP, DNAJB6, CAPN3, TTN, CAV3, SGCG, SGCA, SGCB, SGCD*
PINAmin2	Muscular dystrophy limb-girdle type	*TCAP, CAPN3, TTN*
PINAmin2	Muscular dystrophy limb-girdle type	*SGCG, SGCB, SGCA, SGCD*
PINA	Muscular dystrophy-dystroglycanopathy (congenital with brain and eye anomalies) type	*POMT2, POMT1*
PINA	Muscular dystrophy-dystroglycanopathy (congenital with mental retardation) type	*POMT2, POMT1*
PINA	Muscular dystrophy-dystroglycanopathy (limb-girdle) type	*POMT2, POMT1*
PINA	Myasthenic syndrome congenital associated with acetylcholine receptor deficiency	*CHRNB1, MUSK, RAPSN*
PINA	Myasthenic syndrome fast-channel congenital	*CHRND, CHRNA1, CHRNE*
PINAmin2	Myasthenic syndrome fast-channel congenital	*CHRNA1, CHRND*
PINA	Myasthenic syndrome slow-channel congenital	*CHRND, CHRNA1, CHRNE*
PINAmin2	Myasthenic syndrome slow-channel congenital	*CHRNA1, CHRND*
PINA	Mycobacterial infection atypical familial disseminated	*STAT1, IFNGR1*
PINAmin2	Mycobacterial infection atypical familial disseminated	*STAT1, IFNGR1*
PINA	Myopathy congenital with fiber-type	*ACTA1, TPM3*
PINA	Nasu-Hakola disease	*TYROBP, TREM2*
PINA	Nephrolithiasis/osteoporosis hypophosphatemic	*SLC9A3R1, SLC34A1*
PINA	Nephrotic syndrome type	*NPHS1, NPHS2*
PINA	Neuropathy distal hereditary motor type	*HSPB1, HSPB8*
PINAmin2	Neuropathy distal hereditary motor type	*HSPB1, HSPB8*
PINA	Neuropathy hereditary sensory and autonomic type	*SPTLC1, SPTLC2*
PINA	Noonan syndrome	*SOS1, PTPN11*
PINA	Noonan syndrome	*NRAS, KRAS, RAF1, BRAF*
PINAmin2	Noonan syndrome	*SOS1, PTPN11*
PINAmin2	Noonan syndrome	*RAF1, BRAF, KRAS, NRAS*
PINA	Omenn syndrome	*RAG1, RAG2*
PINA	Orofacial cleft	*SUMO1, MSX1, TP63*
PINA	Osteogenesis imperfecta type	*COL1A1, BMP1, COL1A2*
PINAmin2	Osteogenesis imperfecta type	*COL1A1, COL1A2*
PINA	Osteopetrosis autosomal recessive	*TNFSF11, TNFRSF11A*

Network	Generalised disease term	Causal genes connected in network
PINA	Osteopetrosis autosomal recessive	*CLCN7, OSTM1*
PINA	Osteosarcoma somatic	*CHEK2, RB1*
PINA	Ovarian cancer somatic	*ERBB2, CTNNB1*
PINA	Ovarioleukodystrophy	*EIF2B2, EIF2B5, EIF2B4*
PINA	Pachyonychia congenita type	*KRT17, KRT6A*
PINA	Pancreatic cancer	*TP53, BRCA2*
PINAmin2	Pancreatic cancer	*TP53, BRCA2*
PINA	Paragangliomas	*SDHA, SDHB*
PINAmin2	Paragangliomas	*SDHB, SDHA*
PINA	Parkinson disease	*HTRA2, EIF4G1*
PINA	Parkinson disease	*SNCA, LRRK2*
PINA	Persistent Mullerian duct syndrome type	*AMH, AMHR2*
PINA	Pick disease	*MAPT, PSEN1*
PINA	Pituitary hormone deficiency combined	*PROP1, HESX1*
PINA	Pontocerebellar hypoplasia type	*TSEN2, TSEN54, TSEN34*
PINA	Porencephaly	*COL4A1, COL4A2*
PINAmin2	Porencephaly	*COL4A1, COL4A2*
PINA	Propionicacidemia	*PCCB, PCCA*
PINA	Pseudohypoaldosteronism type	*SCNN1B, SCNN1A, SCNN1G*
PINA	Renal tubular dysgenesis	*AGT, AGTR1*
PINA	Retinitis pigmentosa	*ABCA4, CNGB1, PRPH2, PRPH*
PINA	Retinitis pigmentosa digenic	*PRPH2, PRPH, ROM1*
PINA	Retinitis punctata albescens	*PRPH2, PRPH*
PINA	Roussy-Levy syndrome	*PMP22, MPZ*
PINA	Severe combined immunodeficiency B cell-negative	*RAG1, RAG2*
PINA	Severe combined immunodeficiency T cell-negative B-cell/natural killer-cell positive	*CD3D, CD3E*
PINAmin2	Severe combined immunodeficiency T cell-negative B-cell/natural killer-cell positive	*CD3D, CD3E*
PINA	Sitosterolemia	*ABCG8, ABCG5*
PINAmin2	Sitosterolemia	*ABCG8, ABCG5*
PINA	Spastic paraplegia autosomal recessive	*AP4E1, AP4M1, AP4S1, AP4B1*
PINAmin2	Spastic paraplegia autosomal recessive	*AP4E1, AP4B1*
PINA	Spherocytosis type	*SPTA1, SPTB*
PINA	Spherocytosis type	*SLC4A1, ANK1*
PINAmin2	Spherocytosis type	*SPTA1, SPTB*
PINA	Spinocerebellar ataxia	*ATXN1, ATXN2*
PINA	Stickler syndrome type	*COL2A1, COL9A2*
PINA	Surfactant metabolism dysfunction pulmonary	*CSF2RB, CSF2RA*
PINAmin2	Surfactant metabolism dysfunction pulmonary	*CSF2RA, CSF2RB*
PINA	Telangiectasia hereditary hemorrhagic type	*ENG, ACVRL1*
PINA	Thrombocythemia	*JAK2, MPL, THPO*
PINAmin2	Thrombocythemia	*THPO, MPL*
PINA	Thrombophilia dysfibrinogenemic	*FGB, FGG*
PINAmin2	Thrombophilia dysfibrinogenemic	*FGB, FGG*
PINA	Thyroid carcinoma papillary	*TRIM24, TRIM33*
PINA	Treacher Collins syndrome	*POLR1C, POLR1D*
PINAmin2	Treacher Collins syndrome	*POLR1C, POLR1D*

**Network**	**Generalised disease term**	**Causal genes connected in network**
PINA	Trichothiodystrophy	*ERCC2, ERCC3*
PINAmin2	Trichothiodystrophy	*ERCC3, ERCC2*
PINA	UV-sensitive syndrome	*ERCC6, ERCC8*
PINAmin2	UV-sensitive syndrome	*ERCC8, ERCC6*
PINA	Usher syndrome type	*MYO7A, CDH23, DFNB31, USH1C, USH1G*
PINAmin2	Usher syndrome type	*CDH23, USH1C*
PINA	Ventricular septal defect	*GATA4, NKX2-5*
PINAmin2	Ventricular septal defect	*GATA4, NKX2-5*
PINA	Waardenburg syndrome type	*EDNRB, EDN3*
PINA	Waardenburg syndrome type	*SOX10, PAX3, MITF*
PINAmin2	Waardenburg syndrome type	*PAX3, SOX10*
PINA	Warburg micro syndrome	*RAB3GAP2, RAB3GAP1*
PINA	Xeroderma pigmentosum group	*ERCC2, ERCC3, XPA, XPC, ERCC5, ERCC4*
PINAmin2	Xeroderma pigmentosum group	*ERCC3, ERCC2, ERCC5*

List of all disease subnetworks identified in the PINA and PINAmin2 networks. Note that generalised disease terms have had disease “type” or “group” designations removed.

Table S2 – Performance of intersection filtering method using various parameters

(a) Performance based on disease subnetworks from PINAmin2

**Autosomal Dominant Mode**												
**Variant effect filter**	**Novel**	**Novel**	**Novel**	**Novel**	**Novel**	**Novel**	**Rare**	**Rare**	**Rare**	**Rare**	**Rare**	**Rare**
**Frequency filter**	**Altering**	**Altering**	**Altering**	**Trunc.**	**Trunc.**	**Trunc.**	**Altering**	**Altering**	**Altering**	**Trunc.**	**Trunc.**	**Trunc.**
**Control exomes filter**	**None**	**5 ctrls**	**10 ctrls**	**None**	**5 ctrls**	**10 ctrls**	**None**	**5 ctrls**	**10 ctrls**	**None**	**5 ctrls**	**10 ctrls**
Average # genes per exome with variant(s) after filtering	759.9	114.0	161.8	328.9	40.1	52.6	844.3	137.4	212.7	340.9	49.6	63.3
Disease gene ranked #1	0	309	177	0	20	4	0	293	188	0	21	4
Disease gene ranked #1-2	0	42	16	0	0	0	0	42	18	0	0	0
Disease gene ranked #1-3	0	4	1	0	0	0	0	3	1	0	0	0
Average # disease genes ranked in top ten	0.002	0.757	**0.776**	0.040	0.174	0.055	0.002	0.762	0.657	0.040	0.207	0.061
**Autosomal Recessive Mode**												
**Variant effect filter**	**Novel**	**Novel**	**Novel**	**Novel**	**Novel**	**Novel**	**Rare**	**Rare**	**Rare**	**Rare**	**Rare**	**Rare**
**Frequency filter**	**Altering**	**Altering**	**Altering**	**Trunc.**	**Trunc.**	**Trunc.**	**Altering**	**Altering**	**Altering**	**Trunc.**	**Trunc.**	**Trunc.**
**Control exomes filter**	**None**	**5 ctrls**	**10 ctrls**	**None**	**5 ctrls**	**10 ctrls**	**None**	**5 ctrls**	**10 ctrls**	**None**	**5 ctrls**	**10 ctrls**
Average # genes per exome with variant(s) after filtering	485.0	19.7	31.3	218.2	8.4	15.1	493.3	23.8	35.9	218.9	8.8	15.6
Disease gene ranked #1	0	387	181	0	8	2	0	500	255	0	8	2
Disease gene ranked #1-2	0	68	12	0	0	0	0	124	26	0	0	0
Disease gene ranked #1-3	0	9	3	0	0	0	0	10	3	0	0	0
Average # disease genes ranked in top ten	0.000	1.198	0.733	0.000	0.136	0.034	0.000	**1.491**	0.969	0.000	0.136	0.041
**NeutralMode**												
**Variant effect filter**	**Novel**	**Novel**	**Novel**	**Novel**	**Novel**	**Novel**	**Rare**	**Rare**	**Rare**	**Rare**	**Rare**	**Rare**
**Frequency filter**	**Altering**	**Altering**	**Altering**	**Trunc.**	**Trunc.**	**Trunc.**	**Altering**	**Altering**	**Altering**	**Trunc.**	**Trunc.**	**Trunc.**
**Control exomes filter**	**None**	**5 ctrls**	**10 ctrls**	**None**	**5 ctrls**	**10 ctrls**	**None**	**5 ctrls**	**10 ctrls**	**None**	**5 ctrls**	**10 ctrls**
Average # genes per exome with variant(s) after filtering	1107.1	113.7	161.0	522.3	40.5	53.0	1190.8	136.6	211.5	534.5	50.1	63.6
Disease gene ranked #1	0	339	211	0	25	5	0	342	221	0	29	6

Disease gene ranked #1-2	0	49	21	0	0	0	0	54	29	0	0	0
Disease gene ranked #1-3	0	2	2	0	0	0	0	5	1	0	0	0
Average # disease genes ranked in top ten	0.000	0.830	**0.860**	0.000	0.191	0.069	0.000	0.806	0.744	0.000	0.231	0.071

(b) Performance based on disease subnetworks from PINA

Autosomal Dominant Mode												
**Variant effect filter**	**Novel**	**Novel**	**Novel**	**Novel**	**Novel**	**Novel**	**Rare**	**Rare**	**Rare**	**Rare**	**Rare**	**Rare**
**Frequency filter**	**Altering**	**Altering**	**Altering**	**Trunc.**	**Trunc.**	**Trunc.**	**Altering**	**Altering**	**Altering**	**Trunc.**	**Trunc.**	**Trunc.**
**Control exomes filter**	**None**	**5 ctrls**	**10 ctrls**	**None**	**5 ctrls**	**10 ctrls**	**None**	**5 ctrls**	**10 ctrls**	**None**	**5 ctrls**	**10 ctrls**
Average # genes per exome with variant(s) after filtering	759.9	114.0	161.8	328.9	40.1	52.6	844.3	137.4	212.7	340.9	49.6	63.3
Disease gene ranked #1	0	284	163	0	20	5	0	299	199	0	25	5
Disease gene ranked #1-2	0	36	13	0	0	0	0	31	13	0	0	0
Disease gene ranked #1-3	0	3	0	0	0	0	0	1	0	0	0	0
Average # disease genes ranked in top ten	0.002	0.727	0.744	0.021	0.193	0.053	0.002	**0.756**	0.647	0.021	0.234	0.058
**Autosomal Recessive Mode**												
**Variant effect filter**	**Novel**	**Novel**	**Novel**	**Novel**	**Novel**	**Novel**	**Rare**	**Rare**	**Rare**	**Rare**	**Rare**	**Rare**
**Frequency filter**	**Altering**	**Altering**	**Altering**	**Trunc.**	**Trunc.**	**Trunc.**	**Altering**	**Altering**	**Altering**	**Trunc.**	**Trunc.**	**Trunc.**
**Control exomes filter**	**None**	**5 ctrls**	**10 ctrls**	**None**	**5 ctrls**	**10 ctrls**	**None**	**5 ctrls**	**10 ctrls**	**None**	**5 ctrls**	**10 ctrls**
Average # genes per exome with variant(s) after filtering	485.0	19.7	31.3	218.2	8.4	15.1	493.3	23.8	35.9	218.9	8.8	15.6
Disease gene ranked #1	0	367	161	0	7	2	0	496	246	0	8	3
Disease gene ranked #1-2	0	82	16	0	0	0	0	138	27	0	0	0
Disease gene ranked #1-3	0	4	0	0	0	0	0	11	0	0	0	0
Average # disease genes ranked in top ten	0.000	1.227	0.739	0.000	0.153	0.047	0.000	**1.466**	0.960	0.000	0.181	0.053
**NeutralMode**												
**Variant effect filter**	**Novel**	**Novel**	**Novel**	**Novel**	**Novel**	**Novel**	**Rare**	**Rare**	**Rare**	**Rare**	**Rare**	**Rare**
**Frequency filter**	**Altering**	**Altering**	**Altering**	**Trunc.**	**Trunc.**	**Trunc.**	**Altering**	**Altering**	**Altering**	**Trunc.**	**Trunc.**	**Trunc.**

Control exomes filter	None	5 ctrls	10 ctrls	None	5 ctrls	10 ctrls	None	5 ctrls	10 ctrls	None	5 ctrls	10 ctrls
Average # genes per exome with variant(s) after filtering	1107.1	113.7	161.0	522.3	40.5	53.0	1190.8	136.6	211.6	534.5	50.1	63.7
Disease gene ranked #1	0	308	192	0	24	7	0	329	239	0	29	7
Disease gene ranked #1-2	0	51	18	0	0	0	0	37	22	0	0	0
Disease gene ranked #1-3	0	5	0	0	0	0	0	1	0	0	0	0
Average # disease genes ranked in top ten	0.000	0.803	**0.847**	0.000	0.216	0.074	0.000	0.816	0.747	0.000	0.260	0.075

Results are based on 1,000 tests. “Disease gene ranked *x*” = number of 1,000 simulations in which a spiked disease gene ranked in all of the top *x* positions.

Table S3 – Performance of BioGranat-IG method using various parameters

(a) Performance based on disease subnetworks from PINAmin2 (high-coverage disease subnetworks)

**Autosomal Dominant Mode**				
**Search algorithm**	**Triplet search**	**Triplet search**	**Min/multi distance**	**Min/multi distance**
**Network**	**PINAmin2**	**PINAmin2_d50**	**PINAmin2**	**PINAmin2_d50**
Average # disease genes returned	N/A	1.445	0.970	0.816
Average # disease genes returned in top ten	N/A	**1.385**	0.387	0.444
# scenarios in which all three disease genes returned in top ten	N/A	260	25	70
**Autosomal Recessive Mode**				
**Search algorithm**	**Triplet search**	**Triplet search**	**Min/multi distance**	**Min/multi distance**
**Network**	**PINAmin2**	**PINAmin2_d50**	**PINAmin2**	**PINAmin2_d50**
Average # disease genes returned	N/A	2.413	1.976	2.134
Average # disease genes returned in top ten	N/A	**2.406**	0.006	1.721
# scenarios in which all three disease genes returned in top ten	N/A	597	2	438
**Neutral Mode**				
**Search algorithm**	**Triplet search**	**Triplet search**	**Min/multi distance**	**Min/multi distance**
**Network**	**PINAmin2**	**PINAmin2_d50**	**PINAmin2**	**PINAmin2_d50**
Average # disease genes returned	N/A	1.471	1.020	0.874
Average # disease genes returned in top ten	N/A	**1.409**	0.400	0.475
# scenarios in which all three disease genes returned in top ten	N/A	263	32	74

(b) Performance based on disease subnetworks from PINA (low-coverage disease subnetworks)

**Autosomal Dominant Mode**				
**Search algorithm**	**Triplet search**	**Triplet search**	**Min/multi distance**	**Min/multi distance**
**Network**	**PINAmin2**	**PINAmin2_d50**	**PINAmin2**	**PINAmin2_d50**
Average # disease genes returned	N/A	1.049	0.711	0.557
Average # disease genes returned in top ten	N/A	**0.994**	0.300	0.309
# scenarios in which all three	N/A	131	10	37

disease genes returned in top ten				
**Autosomal Recessive Mode**				
**Search algorithm**	**Triplet search**	**Triplet search**	**Min/multi distance**	**Min/multi distance**
**Network**	**PINAmin2**	**PINAmin2_d50**	**PINAmin2**	**PINAmin2_d50**
Average # disease genes returned	N/A	1.701	1.517	1.448
Average # disease genes returned in top ten	N/A	**1.691**	0.013	1.178
# scenarios in which all three disease genes returned in top ten	N/A	260	4	267
**Neutral Mode**				
**Search algorithm**	**Triplet search**	**Triplet search**	**Min/multi distance**	**Min/multi distance**
**Network**	**PINAmin2**	**PINAmin2_d50**	**PINAmin2**	**PINAmin2_d50**
Average # disease genes returned	N/A	1.083	0.767	0.594
Average # disease genes returned in top ten	N/A	**1.045**	0.336	0.334
# scenarios in which all three disease genes returned in top ten	N/A	131	16	43

Results are based on 1,000 tests. N/A indicates results could not be obtained for triplet search algorithm in the full PINAmin2 network.

Table S4 – Ability of all methods to recover disease subnetworks under varying levels of genetic heterogeneity

	Average # disease genes ranked in top ten				Average # disease genes ranked in top ten				Average # disease genes ranked in top ten			
	**Autosomal Dominant Mode**				**Autosomal Recessive Mode**				**Neutral Mode**			
	Uncaptured heterogeneity				Uncaptured heterogeneity				Uncaptured heterogeneity			
	20%	40%	60%	80%	20%	40%	60%	80%	20%	40%	60%	80%
**Balanced captured Heterogeneity**												
2-gene disease subnetworks:												
HetRank	1.362	1.118	0.772	0.276	1.873	1.756	1.514	0.790	1.398	1.159	0.801	0.277
HetRank (pre-network)	1.299	0.986	0.652	0.208	1.817	1.685	1.355	0.620	1.323	1.029	0.677	0.226
Intersection filtering	1.236	1.074	0.707	0.176	1.810	1.657	1.236	0.443	1.235	1.096	0.750	0.193
BioGranat-IG	0.887	0.815	0.622	0.190	1.270	1.232	1.072	0.534	0.884	0.825	0.633	0.212
3-gene disease subnetworks:												
HetRank	1.707	1.288	0.740	0.220	2.659	2.416	1.922	0.874	1.777	1.353	0.785	0.238
HetRank (pre-network)	1.203	0.825	0.453	0.141	2.389	2.004	1.399	0.527	1.260	0.873	0.493	0.146
Intersection filtering	1.400	1.011	0.500	0.110	2.311	1.814	1.096	0.313	1.468	1.068	0.552	0.128
BioGranat-IG	1.368	1.158	0.798	0.187	1.925	1.800	1.475	0.613	1.376	1.211	0.840	0.232
4-gene disease subnetworks:												
HetRank	1.907	1.322	0.708	0.198	3.351	3.000	2.277	0.895	1.971	1.395	0.750	0.209
HetRank (pre-network)	1.067	0.701	0.375	0.102	2.667	2.034	1.256	0.418	1.130	0.749	0.411	0.114
Intersection filtering	1.326	0.826	0.352	0.066	2.420	1.692	0.896	0.206	1.424	0.902	0.391	0.068
BioGranat-IG	1.678	1.386	0.831	0.187	2.336	2.159	1.696	0.592	1.688	1.436	0.898	0.213
**Unbalanced captured heterogeneity**												
2-gene disease subnetworks:												
HetRank	1.282	1.070	0.727	0.285	1.720	1.619	1.421	0.789	1.301	1.111	0.758	0.296
HetRank (pre-network)	1.108	0.890	0.629	0.238	1.631	1.459	1.181	0.639	1.137	0.925	0.652	0.258
Intersection filtering	0.988	0.849	0.633	0.249	1.538	1.354	1.072	0.520	1.002	0.861	0.663	0.280
BioGranat-IG	0.810	0.754	0.611	0.211	1.213	1.170	1.038	0.534	0.810	0.754	0.623	0.235
3-gene disease subnetworks:												
HetRank	1.643	1.252	0.747	0.229	2.574	2.357	1.883	0.867	1.694	1.310	0.786	0.243
HetRank (pre-network)	1.120	0.814	0.472	0.162	2.235	1.886	1.342	0.553	1.164	0.858	0.512	0.174
Intersection filtering	1.230	0.906	0.494	0.123	2.117	1.693	1.091	0.349	1.267	0.961	0.533	0.139

	Average # disease genes ranked in top ten				Average # disease genes ranked in top ten				Average # disease genes ranked in top ten			
	**Autosomal Dominant Mode**				**Autosomal Recessive Mode**				**Neutral Mode**			
	Uncaptured heterogeneity				Uncaptured heterogeneity				Uncaptured heterogeneity			
	20%	40%	60%	80%	20%	40%	60%	80%	20%	40%	60%	80%
BioGranat-IG	1.277	1.117	0.741	0.187	1.866	1.761	1.467	0.644	1.302	1.130	0.794	0.217
4-gene disease subnetworks:												
HetRank	1.754	1.264	0.698	0.203	3.152	2.835	2.172	0.873	1.833	1.341	0.763	0.221
HetRank (pre-network)	0.983	0.690	0.395	0.122	2.278	1.803	1.202	0.462	1.037	0.742	0.419	0.134
Intersection filtering	1.015	0.708	0.390	0.088	1.982	1.487	0.923	0.293	1.073	0.743	0.424	0.101
BioGranat-IG	1.430	1.153	0.717	0.162	2.199	2.035	1.556	0.602	1.462	1.207	0.737	0.180

All results are based on 1,000 tests.

Table S5 – Improved ability to recover disease subnetworks under varying levels of genetic heterogeneity using network-informed HetRank approach relative to simple intersection filtering

	**Avg. increase: # disease genes ranked in top ten**				**Increase: # tests in which all disease genes rank in top ten**			
	Uncaptured heterogeneity				Uncaptured heterogeneity			
	20%	40%	20%	40%	20%	40%	20%	40%
**Autosomal Dominant Mode**								
Balanced captured heterogeneity:								
2-gene disease subnetworks	0.126	0.044	0.065	0.100	108	77	104	55
3-gene disease subnetworks	0.307	0.277	0.240	0.110	187	161	74	10
4-gene disease subnetworks	0.581	0.496	0.356	0.132	100	52	18	2
Unbalanced captured heterogeneity:								
2-gene disease subnetworks	0.294	0.221	0.094	0.036	186	186	117	41
3-gene disease subnetworks	0.413	0.346	0.253	0.106	187	141	67	10
4-gene disease subnetworks	0.739	0.556	0.308	0.115	83	37	16	2
**Autosomal Recessive Mode**								
Balanced captured heterogeneity:								
2-gene disease subnetworks	0.063	0.099	0.278	0.347	61	95	238	187
3-gene disease subnetworks	0.348	0.602	0.826	0.561	286	405	324	88
4-gene disease subnetworks	0.931	1.308	1.381	0.689	443	353	185	32
Unbalanced captured heterogeneity:								
2-gene disease subnetworks	0.182	0.265	0.349	0.269	172	261	321	162
3-gene disease subnetworks	0.457	0.664	0.792	0.518	341	403	300	86
4-gene disease subnetworks	1.170	1.348	1.249	0.580	381	294	150	24
**Neutral Mode**								
Balanced captured heterogeneity:								
2-gene disease subnetworks	0.163	0.063	0.051	0.084	131	81	97	50
3-gene disease subnetworks	0.309	0.285	0.233	0.110	202	169	82	12
4-gene disease subnetworks	0.547	0.493	0.359	0.141	108	59	22	2
Unbalanced captured heterogeneity:								
2-gene disease subnetworks	0.299	0.250	0.095	0.016	187	194	110	42
3-gene disease subnetworks	0.427	0.349	0.253	0.104	198	157	73	11
4-gene disease subnetworks	0.760	0.598	0.339	0.120	95	43	16	1

All results are based on 1,000 tests.

Table S6 – Summary of top prioritised genes in 13 Adams-Oliver syndrome exomes

(a) Top prioritised genes using PINA network

Position	Gene	Evidence for disease involvement
1	*XRCC6*	Novel truncating or splicing variants in direct neighbours *NOTCH1* (2 exomes), *XRCC5*, *ILVBL* and *HERPUD1* (1 exome each)
2	*GSK3B*	Novel truncating or splicing variants in direct neighbours *FAM83D*, *GNB2*, *KIF5B*, *NOTCH1* and *UBXN6* (1 exome each)
3	*MAPK6*	Novel truncating or splicing variant in 1 exome (and no control exomes); novel altering variant in 1 exome (and 1 control exome); novel truncating or splicing variants in direct neighbours *NDUFS6* and *NEURL4* (1 exome each)
4	*PLK1*	Novel truncating or splicing variants in direct neighbours *CTBP1*, *MAPK6* and *NINL* (1 exome each); novel altering variants in direct neighbours *ACTL6B* and *CHEK2* (1 exome each)
5	*PHYH*	Novel truncating or splicing variants in direct neighbours *NOTCH1* (2 exomes), *CRIM1* and *GNB2* (1 exome each); novel synonymous variant in direct neighbour *MOAP1* (1 exome)
6	*NINL*	Novel truncating or splicing variants in 2 exomes (and no control exomes); novel truncating or splicing variant in direct neighbour *EZH2* (1 exome)
7	*VPS54*	Novel truncating or splicing variant in 1 exome (and no control exomes); rare truncating or splicing variant in 1 exome (and no control exomes); novel truncating or splicing variant in indirect neighbour *UBE2Z* (1 exome)
8	*TFAP4*	Novel truncating or splicing variants in direct neighbours *CTBP1*, *UBP1* and *USF2* (1 exome each); novel altering variant in direct neighbours *HMGB3* and *THYN1* (1 exome each)
9	*TPSB2*	Novel truncating or splicing variants in 2 exomes (and no control exomes)
10	*MCM10*	Novel truncating or splicing variants in direct neighbours *NINL* (2 exomes) and *CUL4A* (1 exome); rare truncating or splicing variant in direct neighbour *CDKN1A* (1 exome)
12	***NOTCH1***	Novel truncating or splicing variants in 2 exomes (and no control exomes)
=187	***DLL4***	Novel missense variant in 1 exome (and 1 control exome); novel truncating or splicing variants in direct neighbour *NOTCH1* (2 exomes)

(b) Top prioritised genes using PINAmin2 network

Position	Gene	Evidence for disease involvement
1	*TPSB2*	Novel truncating or splicing variants in 2 exomes (and no control exomes)
2	*NINL*	Novel truncating or splicing variants in 2 exomes (and no control exomes)
3	***NOTCH1***	Novel truncating or splicing variants in 2 exomes (and no control exomes)
4	*DCUN1D5*	Novel truncating or splicing variants in 2 exomes (and no control exomes)
5	*GNB2*	Novel truncating or splicing variant in 1 exome (and no control exomes); rare truncating or splicing variants in 2 exomes (one of which has its score bettered in 1 control exome)
6	*PPEF2*	Novel truncating or splicing variant in 1 exome (and no control exomes); rare truncating or splicing variant in 1 exome (and no control exomes)
7	*EP300*	Novel truncating or splicing variants in direct neighbours *CTBP1*, *ETS2* and *NOTCH1* (1 exome each); novel synonymous variant in direct neighbour *STAT1* (1 exome)
8	*LRIT1*	Novel truncating or splicing variant in 1 exome (and no control exomes); rare truncating or splicing variant in 1 exome (and no control exomes)

Position	Gene	Evidence for disease involvement
9	*VPS54*	Novel truncating or splicing variant in 1 exome (and no control exomes); rare truncating or splicing variant in 1 exome (and no control exomes)
10	*RPE*	Novel truncating or splicing variant in 1 exome (and no control exomes); novel synonymous variant in 1 exome (and no control exomes)
1047	***DLL4***	Novel missense variant in 1 exome (and 1 control exome)

Table S7 – Ability to prioritise Adams-Oliver syndrome genes with additional disease-causing variants

Variants added	***NOTCH1*** **rank**	***DLL4*** **rank**
No added variants	12	=187
DLL4 variant in 1 additional exome	12	35
DLL4 variant in 2 additional exomes	13	7
NOTCH1 variant in 1 additional exome	1	47
NOTCH1 variant in 2 additional exomes	1	24
NOTCH1 variant in 3 additional exomes	1	20
NOTCH1 variant in 4 additional exomes	1	20
